# Combined Transcriptomic and Metabolomic Analysis Reveals an Ethylene‐Activated Regulatory Model on Monoterpenoid Indole Alkaloid Biosynthesis in *Catharanthus roseus*

**DOI:** 10.1111/pbi.70343

**Published:** 2025-09-08

**Authors:** Bofu Deng, Qing Miao, Chaoqin Ou, Yuanbing Pan, Hang Liu, Xueqing Fu, Ling Li, Yuliang Wang, Kexuan Tang, Qifang Pan

**Affiliations:** ^1^ Frontiers Science Center for Transformative Molecules, Joint International Research Laboratory of Metabolic & Developmental Sciences, Plant Biotechnology Research Center, SJTU‐Fudan‐Nottingham Plant Biotechnology R&D Center, School of Agriculture and Biology Shanghai Jiao Tong University Shanghai China; ^2^ School of Biological Sciences The University of Hong Kong Hong Kong Hong Kong; ^3^ Yazhouwan National Laboratory Sanya Hainan China

**Keywords:** *Catharanthus roseus*, EIN3/EIL transcription factors, ethylene signal, monoterpenoid indole alkaloids, omics analysis

## Abstract

*Catharanthus roseus* contains nearly 200 bioactive monoterpenoid indole alkaloids (MIAs) that are effective in treating cancer and other diseases. Ethylene plays a significant role in enhancing MIA biosynthesis, and we have found that it greatly induces the accumulation of anhydrovinblastine. However, the regulatory mechanisms underlying this process are not yet fully understood. In this study, a comprehensive analysis of the metabolome and transcriptome of *C. roseus* was conducted to identify two EIN3/EIL transcription factors, CrEIN3 and CrEIL1, which act as key components mediating the ethylene signal to upregulate MIA biosynthesis. Both CrEIN3 and CrEIL1 were found to upregulate the expression of MIA biosynthetic genes and the activator gene ORCA3, while repressing the expression of repressor genes GBF1 and ZCT1, resulting in increased vinblastine production in *C. roseus* . CrEIN3 directly binds to the SGD promoter, while CrEIL1 interacts with JA‐induced BIS2 to enhance upregulation of the iridoid pathway, thereby further promoting downstream MIA biosynthesis and strengthening the accumulation of bisindole MIAs. Our findings reveal an ethylene‐activated regulatory model consisting of CrEIN3 and CrEIL1 that integrates JA‐induced BIS2 to cooperatively regulate MIA production in *C. roseus* , shedding light on the mechanism of ethylene signal regulating MIA biosynthesis. This research provides a foundation for understanding plant hormone regulation of alkaloid metabolism, which will contribute to future efforts in developing high‐yielding MIAs in plant or yeast‐based platforms.

## 1 Introduction

*Catharanthus roseus* is a well‐studied medicinal plant that produces almost 200 monoterpenoid indole alkaloids (MIAs) (De Luca et al. 2014). Some of these compounds have been found to have strong pharmacological activities and are widely used in the clinical treatment of cancer (Almagro et al. 2015). For example, vincristine and vinblastine are important constituents used in chemotherapies for Hodgkin lymphoma, lymphosarcoma and neuroblastoma (Verma et al. 2017). Other MIAs, such as ajmalicine and serpentine, have been used in the treatment of circulatory system diseases, while vindoline and catharanthine have shown potential as a treatment for diabetes (Van Der Heijden et al. 2004).

To optimise the yield of monoterpenoid indole alkaloids (MIAs) in *C. roseus*, tobacco or yeast cell factories, it is necessary to fully understand the biosynthetic genes and regulatory mechanisms involved in the MIA pathways (Birchfield and McIntosh 2020; Liu et al. 2021; Zhang et al. 2022; Grzech et al. 2023; Salim et al. 2023; Gao et al. 2023). Recent advances in high‐throughput sequencing of the genome and transcriptome, especially single‐cell multi‐omics analyses of *C. roseus* revealing cell‐type‐specific expression, have facilitated the identification of previously unknown enzymatic steps in the downstream MIAs pathway, such as Geissoschizine Synthase 1 and 2 (GS1 and 2), Geissoschizine Oxidase (GO), Redox1 and Redox2, Stemmadenine‐O‐Acetyltransferase (SAT), Precondylocarpine Acetate Synthase (PAS), Dihydroprecondylocarpine Acetate Synthase (DPAS), Catharanthine Synthase/Hydrolase 1 (CS/HL1) or Tabersonine Synthase/Hydrolase 2 (TS/HL2), among others (De Luca et al. 2014; Qu et al. 2015, 2018, 2019; Stavrinides et al. 2016; Tatsis et al. 2017; Caputi et al. 2018; Xu et al. 2023; Li et al. 2023). However, the transcriptional regulation of these genes remains unclear. While significant progress has been made in understanding jasmonate (JA)‐responsive transcriptional regulation in *C. roseus* , particularly the JAZ‐CrMYC2/BIS‐ORCA module that regulates MIA biosynthesis under JA induction, it is important to note that the expression of catharanthine and vindoline pathway genes, such as SGD, Redox1, Redox2, SAT, CS and TS, is not controlled by this module (Patra et al. 2018; Schweizer et al. 2018; Singh et al. 2020, 2021). Therefore, it is likely that the expression of these genes is subject to regulation by other plant hormones.

Ethylene is a phytohormone that affects various aspects of plant development, secondary metabolism and stress responses. Previous studies have demonstrated that ethylene treatment positively influences the transcription and metabolite levels involved in the MIA biosynthesis in *C. roseus* . Exogenous ethylene has been found to effectively enhance MIA biosynthesis in different parts of *C. roseus* , including plants, hairy roots and cell suspensions (Chen et al. 2017). Specifically, ethylene treatments have been shown to promote the accumulation of vinblastine, catharanthine and ajmalicine (Vazquez‐Flota et al. 2009; Pan et al. 2010; Chen et al. 2017). The expression of both CrWRKY1 and CrERF5, which are associated with ethylene signalling, exhibited a strong response (Suttipanta et al. 2011; Pan et al. 2019). Additionally, ethylene treatment induced the expression of HL1 and PRX1 transcripts in the shoots and roots of 8‐day‐old *C. roseus* seedlings (Costa et al. 2008; Fraser et al. 2020). While the molecular mechanism of the ethylene signal transduction pathway has been extensively studied, there are limited reports on ethylene‐induced transcription factors (TFs) regulating MIA biosynthesis. Downstream TFs of the ethylene signalling pathway, such as EIN3/EILs and AP2/ERFs, have garnered attention for their role in regulating secondary metabolism in higher plants (Chao et al. 1997). AP2/ERFs have been implicated in the accumulation of MIAs in *C. roseus* . However, it remains unknown whether EIN3/EILs are involved in MIA biosynthesis.

In this study, we conducted a transcriptome and metabolome analysis of *C. roseus* and identified two EIN3/EILs (CrEIN3 and CrEIL1) as potential regulators of MIA biosynthesis. We cloned and characterised these TFs and found that CrEIN3 positively regulates vinblastine accumulation by binding to the promoter of SGD. Additionally, CrEIL1 was found to interact with BIS2 and activate its transcriptional activation activity. Our findings suggest that both CrEIN3 and CrEIL1 contribute to ethylene signalling and play a role in regulating MIA biosynthesis in *C. roseus* . This study provides insight into the mechanism by which ethylene affects MIA biosynthesis and expands our understanding of the regulatory effect of ethylene on the MIAs metabolic pathway.

## 2 Results

### 2.1 Ethylene and Jasmonate Signals Stimulated the Accumulation of Monoterpenoid Indole Alkaloids and Precursors in *Catharanthus roseus* Plants

**FIGURE 1:**
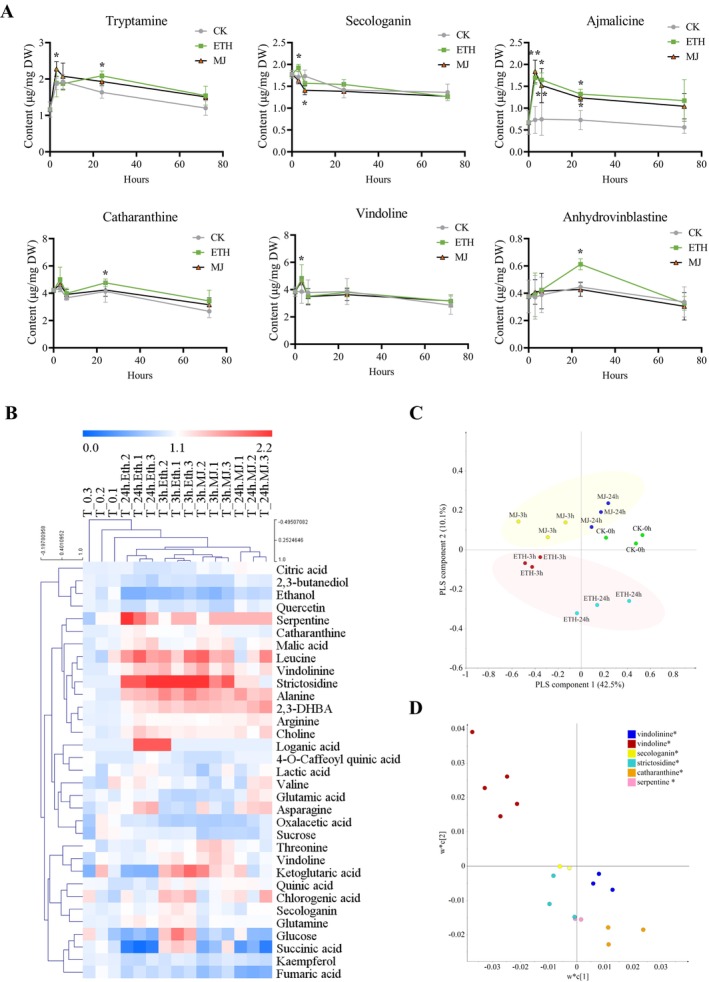
MIA‐targeted analysis and non‐targeted analysis of *Catharanthus roseus* under ethephon (ETH) and methyl jasmonate (MJ) treatment. (A) MIAs contents under ethephon (ETH) and methyl jasmonate (MJ) treatment in *C. roseus* . The error bars represent the means ± SD (standard deviation) from three replicates, and asterisks indicate statistically significant differences compared with the controls using the student's *t*‐test. *p < 0.05. (B) Overview of *C. roseus* metabolome under ETH or MJ treatments based on NMR‐metabolome data. The colour scale (blue to red) represents the fold changes of the metabolite levels at different time points under ETH or JA treatment compared to the control at 0 h. (C) Score plot of partial least square‐discriminant (PLS‐DA) analysis of *C. roseus* leaves under ETH treatment or MJ treatment based on NMR‐metabolome data. (D) Loading blot of *C. roseus* leaves under ETH or MJ treatment based on NMR‐metabolome data.

Metabolite analysis using UPLC‐Q/TOF MS was performed on *C. roseus* leaves at different time points (0, 3, 6, 24 and 72 h) following treatments with ethephon (ETH) and methyl jasmonate (MJ) (Figure 1A). The results showed that both ETH and MJ treatments induced the accumulation of tryptamine and ajmalicine. Additionally, ethylene significantly stimulated the biosynthesis of secologanin, catharanthine and anhydrovinblastine. Particularly, the content of anhydrovinblastine in the ETH‐treated samples at 24 h increased by 65.27% compared to the samples at 0 h, and was much higher than in the controls and MJ‐treated samples, indicating that the ethylene signal plays a vital role in bisindole alkaloid biosynthesis. Moreover, the contents of tryptamine, ajmalicine, and vindoline peaked at 3 h under MJ treatment while the contents of tryptamine, catharanthine, and anhydrovinblastine peaked at 24 h under ETH treatment. Based on these results, time points of 0, 3 and 24 h after ETH or MJ treatments were selected for NMR‐based metabolomic studies and RNA‐seq analysis.

### 2.2 Metabolome Variations Confirms the Response of Monoterpenoid Indole Alkaloids Biosynthesis to Both Jasmonate and Ethylene Signals in *Catharanthus roseus*

To investigate the dynamics of MIA biosynthesis under jasmonate and ethylene signals, NMR‐based metabolomic profiling was conducted on *C. roseus* samples. A combination of 2D‐NMR (J‐resolved and COSY) and previously reported NMR profiling data (Pan et al. 2014; Yang et al. 2009; Kim, Choi, and Verpoorte 2010) was used to elucidate and identify approximately 40 primary and secondary metabolites related to MIA biosynthesis, including two precursors and five MIAs (Figure S1A–C; Table S2). The heatmap revealed that the detected metabolites from individual samples were divided into four groups: I, II, III and IV (Figure 1B). Detected MIAs, such as strictosidine, serpentine, catharanthine and vindolinine, were mainly clustered in group II and responded to both ETH and MJ induction (Figure 1B).

The ^1^H‐NMR data from all samples underwent various multivariate data analyses (MVDA) to illustrate the dynamic variations during the MJ or ETH response period. A score plot of PLS‐DA, validated by permutation test, depicted the separation of all samples into five distinct groups based on PLS components 1 and 2: the 0 h control group, the 3 h‐MJ treated group, the 24 h‐MJ treated group, the 3 h‐ETH treated group, and the 24 h‐ETH treated group (Figure 1C; Figure S1D). The MJ‐treated groups (at 3 and 24 h) were positioned in the upper part of the quadrant, while the ETH‐treated groups (at 3 and 24 h) were located in the lower part, indicating a differential response in MIA biosynthesis to JA and ET signals in *C. roseus* plants (Figure 1C). According to the loading plot, signals corresponding to catharanthine and serpentine were predominantly present in the 24 h‐ETH treated group, suggesting higher levels of these compounds accumulated in the samples 24 h after ETH treatment (Figure 1D). Secologanin signals were observed in the 3 h‐ETH treated group. Signals of strictosidine and vindolinine were detected in both the 3 h‐ETH and 24 h‐ETH treated groups, while vindoline signals were observed in the 3 h‐MJ treated group. Further statistical analysis revealed that, compared to the controls at 0 h, the signal intensity of strictosidine significantly increased 2.08‐fold at 3 h, 1.33‐fold at 24 h under ETH treatment, and 1.25‐fold at 3 h under MJ treatment (Table S4). The signal intensity of vindolinine significantly increased by 36% at 3 h, 32% at 24 h under ETH treatment and 39.3% at 3 h under MJ treatment. The signal intensities of catharanthine and serpentine significantly increased by 15% and 100%, respectively, at 24 h under ETH treatment. The signal intensity of vindoline significantly increased by 29% at 3 h under MJ treatment. No significant changes in MIA levels were observed at 24 h after MeJA treatment (Table S4). The results were consistent with those obtained from LC–MS and heatmap analyses. These findings suggested that MJ treatment led to a rapid accumulation of vindoline over a short time frame, while ETH treatment had a more sustained effect on the accumulation of strictosidine, catharanthine, vindolinine and serpentine.

### 2.3 Co‐Expression Network of Ethylene and Jasmonate‐Mediated Transcriptional and Metabolic Reprogramming

**FIGURE 2:**
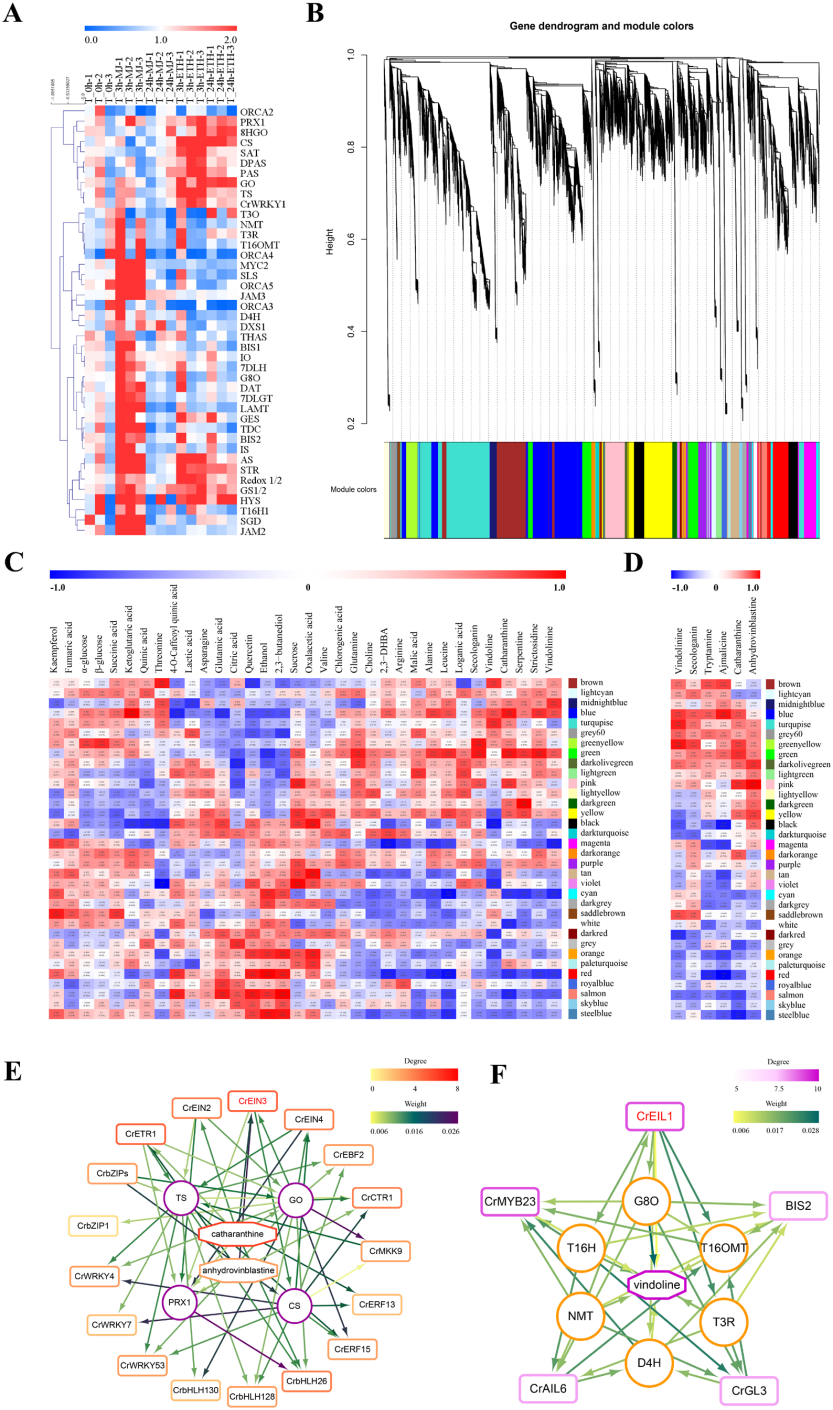
MIAs related metabolome and transcriptome data analysis in *C. roseus* under ETH or MJ treatment. (A) HCL analysis of MIAs related gene transcripts under ETH treatment. The colour scale (blue to red) represents the fold changes of the FPKM values of each gene at different time points under ETH or JA treatment compared to the control at 0 h. (B) Dendrogram showing co‐expression modules (clusters) identified by weighted correlation network analysis (WGCNA) of *C. roseus* under ETH and MJ treatments. (C, D) Heat map showing module‐untargeted metabolites correlations and module‐MIAs correlations, respectively. Each column corresponds to a module indicated by different colours. Each row corresponds to a metabolite or MIA. Red colour indicates a positive correlation between the module and the metabolite. Blue colour indicates a negative correlation. (E, F) Gene regulatory network of anhydrovinblastine and catharanthine from the green and pink modules and gene regulatory network of vindoline from the turquoise module by GENIE3. The colour intensity of the edges represents the weight between two nodes, and the colour variation of the node borders represents the degree of TFs gene. The circles represent enzymes and the rectangular boxes represent transcription factors (TFs).

To investigate the regulatory network of MIAs in response to ethylene and jasmonate signals, RNA‐seq data were collected at three time points (0, 3 and 24 h) following ETH or MJ treatments. After removing adapter sequences, ambiguous reads, and low‐quality reads, an average of 8.98 Gb of clean reads was obtained (Dataset S2). The expression levels, indicated by FPKM values, showed strong correlations among biological replicates. In total, 33 830 genes were identified as differentially expressed under ETH or MJ treatments (Figure S2; Dataset S3). Hierarchical clustering analysis revealed that the MIA‐related genes could be grouped into three categories (Figure 2A). Group I genes were predominantly induced by ETH treatment, including *PRX1, 8‐HGO, CS, SAT, DPAS, PAS, GO, TS* and *CrWRKY1*. Group II genes responded to both ETH and MJ treatments, with notable genes like *AS, STR, Redox1/2, GS1/2, HYS, T16H1* and *SGD*. Group III genes were specifically induced by MJ treatment. Furthermore, ethylene‐induced MIA genes showed sustained upregulation from 3 to 24 h, while jasmonate‐induced MIA genes exhibited a rapid response at 3 h and returned to baseline levels by 24 h, as verified by qPCR (Figure S3A–C).

To further explore the regulation of MIA biosynthesis in response to ethylene and JA signals, we performed weighted gene co‐expression network analysis (WGCNA) to identify co‐expression networks of differentially expressed genes (DEGs) (log2FC > 0.585, FDR < 0.05). A total of 34 co‐expression modules were identified based on similar expression patterns (Figure 2B; Dataset S4). The heatmap analysis of module‐untargeted metabolite correlations revealed that 16 modules were positively correlated with MIAs (Figure 2C; Dataset S5). To confirm these correlations, we analysed the module‐targeted MIA correlations (Figure 2D; Dataset S5). The results showed that the brown module had a significant positive correlation with tryptamine accumulation, while the greenyellow module was strongly correlated with secologanin accumulation. The blue and brown modules were positively correlated with ajmalicine accumulation, while the turquoise and greenyellow modules showed significant correlations with vindoline accumulation. The green and pink modules were highly correlated with catharanthine accumulation, and the pink module exhibited a significant correlation with anhydrovinblastine accumulation.

The key structural genes involved in the MIA biosynthesis pathway were primarily found in the brown, green, pink, blue and turquoise modules. Genes in the brown module were preferentially expressed 3 h after MJ treatment, while those in the green and pink modules exhibited heightened responsiveness to ethylene signal at both 3 and 24 h (Figure S4). Genes in the blue and turquoise modules showed a preference for expression under both MJ and ETH treatments at 3 h (Figure S4). The brown module contained JA‐responsive TF genes related to the upstream MIA pathway, such as *JAZs, MYC2* and *ORCA3*. In the green and pink modules, four MIA downstream structural genes (*CS, TS, GO* and *PRX1*) were identified. Unexpectedly, the turquoise and blue modules, which responded to both JA and ethylene signals, contained a high number of structural genes involved in vindoline and iridoid pathways. Noteworthy genes in the turquoise module included *T16H1/2, T16OMT, T3O, T3R, NMT, D4H, G8O, IO* and *7DLH*.

We established the co‐expression networks of the turquoise, green and pink modules associated with vindoline, catharanthine and anhydrovinblastine, which were key precursors in the production of vinblastine and vincristine (Figure S4B,C; Datasets S6 and S7). In the co‐expression networks of the green and pink modules, a total of 40 TF genes showed significant correlations with the four structural genes (*CS, TS, GO*, and *PRX1*). In the co‐expression network of the turquoise module, 37 TF genes (with modkME > 0.9) were significantly correlated with iridoid and vindoline biosynthetic genes (*G8O, T16H1/2, T16OMT, T3O, T3R, NMT, D4H*).

GENIE3 was then used to predict the gene regulatory network based on these results. In the gene regulatory networks of the green and pink modules, the weight values of the links between 17 TF‐encoding genes and MIA pathway genes were greater than 0.006, including WRKYs, ERFs, bZIPs, bHLHs, and ethylene signal‐responsive proteins (such as EBF2, EIN2, EIN3, EIN4, ETR1 and CTR1) (Figure 2E). Among them, CrEIN3 (CRO_017077), an EIN3/EIL TF, showed a strong regulatory relationship with structural genes (*GO, CS* and *PRX1*) and MIAs (catharanthine and anhydrovinblastine), with an average link weight value of 0.016. In the gene regulatory network of the turquoise module, the weight values of the links between 5 TF‐encoding genes and MIA pathway genes were greater than 0.006, including *EIL1, MYB23, BIS2, AIL6* and *GL3* (Figure 2F). Notably, another EIN3/EIL TF, CrEIL1 (CRO_031898), exhibited a strong regulatory relationship with iridoid and vindoline biosynthetic genes (*T16H, T16OMT, T3R, NMT, D4H, G8O*), with an average link weight value of 0.017.

### 2.4 Identification of CrEIN3 and CrEIL1 as the Key TFs in the Regulation of Ethylene Signals on the Biosynthesis of Monoterpenoid Indole Alkaloids

**FIGURE 3:**
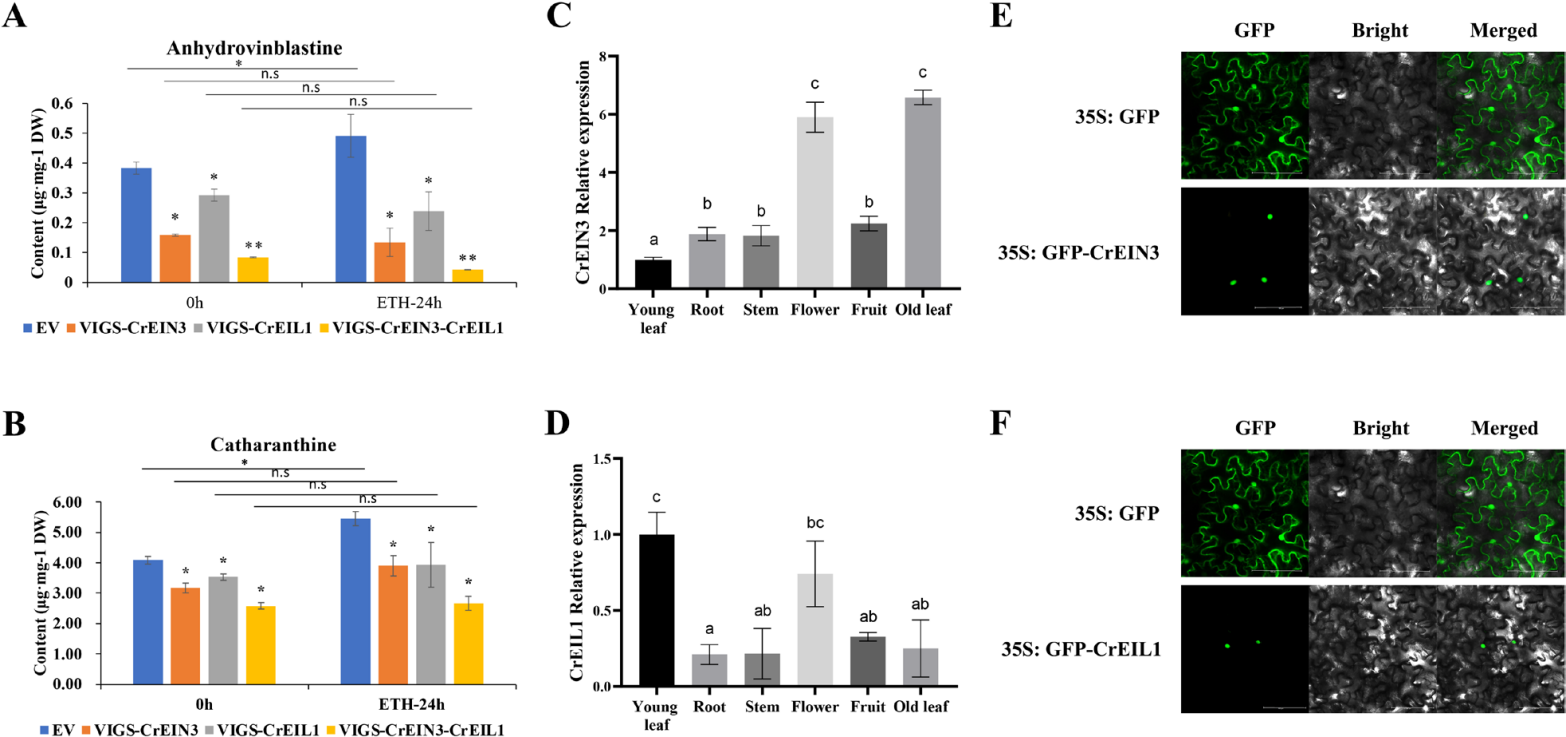
Expression pattern and subcellular localization of CrEIN3 and CrEIL1. (A, B) MIAs contents in *CrEIN3*‐ and *CrEIL1*‐ sienced samples under ETH treatment. *p < 0.05, **p < 0.01, by Student's *t*‐test. (C, D) Relative expression levels of *CrEIN3* and *CrEIL1*, respectively, in young leaf (set to 1), root, stem, flower, seed pod and old leaf of *C. roseus* . Different letters indicate significant difference (*p* < 0.05 by one way ANOVA). (E, F) Subcellular localization of CrEIN3 and CrEIL1 in tobacco leaves. Bars = 100 μm. The error bars represent the means ± SD (standard deviation) from three replicates.

To elucidate the gene functions of CrEIN3 and CrEIL1, we attempted to complement the Arabidopsis ein3eil1 mutant. The introduction of *CrEIN3* or *CrEIL1* cDNA successfully complemented the *ein3einl1* mutant, restoring its response to ethylene (Figure S5). To further investigate the potential roles of CrEIN3 and CrEIL1 as key regulators of MIA biosynthesis in the ethylene signalling pathway, we employed the VIGS method to silence *CrEIN3, CrEIL1*, and both genes simultaneously in *C. roseus* plantlets. We then assessed the levels of catharanthine and anhydrovinblastine in response to ETH treatment. The findings revealed a significant reduction in the accumulation of both catharanthine and anhydrovinblastine in the silenced *C. roseus* plantlets compared to the control group (Figure 3A,B). Furthermore, the ETH treatment failed to induce the accumulation of catharanthine and anhydrovinblastine in the silenced *C. roseus* plantlets, particularly in those where both *CrEIN3* and *CrEIL1* were silenced (Figure 3A,B). These results strongly suggest that CrEIN3 and CrEIL1 play pivotal roles as key components in mediating the ethylene signal to regulate MIA biosynthesis.

The expression patterns of *CrEIN3* and *CrEIL1* in various tissues of *C. roseus* were examined using RT‐qPCR. The analysis revealed that *CrEIN3* exhibited higher expression levels in flowers and old leaves compared to other organs (Figure 3C), while *CrEIL1* displayed higher expression levels in young leaves and flowers (Figure 3D). Subsequently, to investigate the subcellular localization of CrEIN3 and CrEIL1, we utilised laser scanning confocal microscopy to visualise the GFP fluorescence signals of the fusion proteins CrEIN3‐GFP and CrEIL1‐GFP expressed in *N. benthamiana* leaves. Both CrEIN3‐GFP and CrEIL1‐GFP signals were distinctly localised in the nucleus (Figure 3E,F).

### 2.5 CrEIN3 and CrEIL1 Positively Regulates the Biosynthesis of Monoterpenoid Indole Alkaloids

To elucidate the biological functions of CrEIN3 and CrEIL1, we generated both overexpression and VIGS constructs to assess their effects on MIA accumulations in *C. roseus* . The recombinant plasmids pHB‐*CrEIN3/CrEIL1*‐GFP or pTRV2:*CrEIN3/CrEIL1* were introduced into the petals or leaves of *C. roseus* plants using *Agrobacterium tumefaciens* strain GV3101. Western blot analysis showed that the CrEIN3/CrEIL1‐GFP fusion protein was successfully expressed in OE petals (Figure S9). VIGS significantly reduced CrEIN3 gene expression by 47% and CrEIL1 gene expression by 51% in the silenced lines compared to the controls (Figure S6).

**FIGURE 4:**
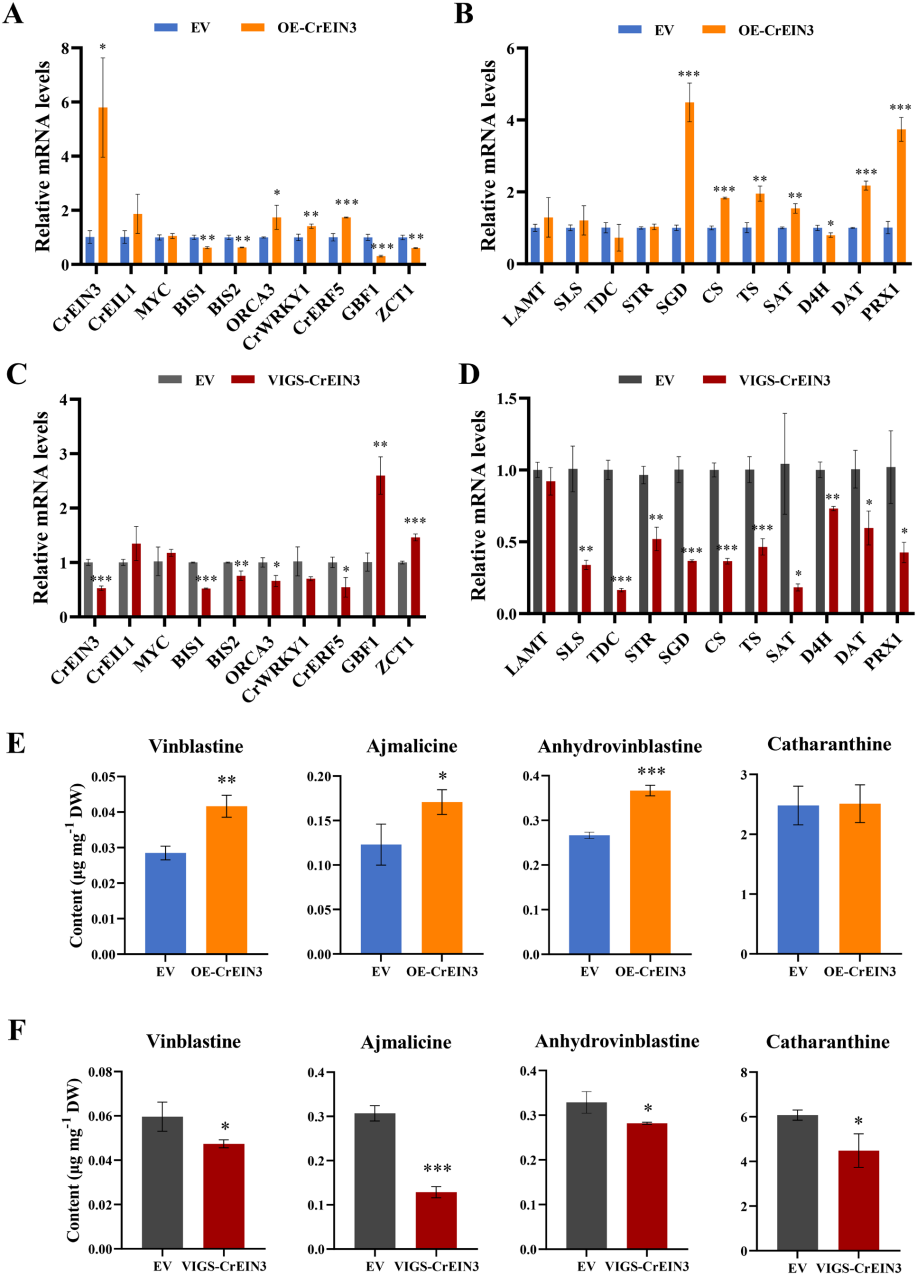
Regulatory effect of CrEIN3 on MIAs biosynthesis in *C. roseus* . (A, B) Gene expression levels of MIAs related genes in *CrEIN3* overexpression lines. (C, D) Gene expression levels of MIAs related genes in *CrEIN3* silencing lines. (E) MIAs contents in *CrEIN3* overexpression lines. EV, empty vector; OE, overexpression. (F) MIAs contents in *CrEIN3* silencing lines. EV, empty vector; VIGS, virus induced gene silence. The error bars represent the means ± SD (standard deviation) from three replicates. Asterisks indicate statistically significant differences using Student's *t*‐test. *p < 0.05, **p < 0.01, ***p < 0.001.

In *CrEIN3*‐OE petals, qRT‐PCR results demonstrated a significant increase in *CrEIN3* expression compared to the controls (Figure 4A). Additionally, expression levels of two positive regulator genes (*ORCA3, CrERF5*) and five key enzyme genes involved in MIA biosynthesis (*SGD, CS, TS, DAT* and *PRX1*) were notably higher than in controls (Figure 4A,B). Conversely, expression levels of two transcriptional repressor genes (*GBF1* and *ZCT1*) were significantly lower than in controls (Figure 4A). Furthermore, levels of vinblastine, anhydrovinblastine, and ajmalicine were significantly higher in CrEIN3‐OE petals than in controls (Figure 4E). In *CrEIN3*‐silenced plants, inhibiting *CrEIN3* expression led to significant decreases in expression of iridoid pathway genes (*SLS*), downstream MIA pathway genes (*STR, SGD, CS, TS, SAT, DAT, PRX1*), and TF genes (*ORCA3* and *CrERF5*) (Figure 4C,D). However, the expression of *GBF1* and *ZCT1* increased significantly. Moreover, in *CrEIN3*‐silenced samples, contents of anhydrovinblastine, vinblastine, ajmalicine, and catharanthine were notably reduced (Figure 4F).

In *CrEIL1*‐OE petals, the expression levels of *CrEIL1, ORCA3, BIS2, CrWRKY1, CrERF5, TDC, STR, SAT, D4H, DAT* and *PRX1* were significantly elevated, while the expression levels of *GBF1* and *ZCT1* were notably lower than in the controls (Figure S10A,B). Conversely, in *CrEIL1*‐silenced plants, inhibiting *CrEIL1* expression led to significant decreases in the expression of iridoid pathway genes (*7DLH, LAMT, SLS*), indole pathway genes (*TDC*), downstream MIA pathway genes (*STR, SGD, SAT, CS, TS, PRX1*), vindoline pathway genes (*D4H, DAT*), and TF genes (*ORCA3, CrWRKY1* and *CrERF5*) (Figure S10C,D). LC/MS results revealed that *CrEIL1*‐OE petals contained higher levels of secologanin, ajmalicine, and vinblastine compared to the controls, while *CrEIL1*‐silenced samples exhibited significant reductions in the contents of secologanin, vindoline, ajmalicine, and vinblastine (Figure S10E,F). Collectively, these results demonstrate that both CrEIN3 and CrEIL1 act as positive regulators in the biosynthesis of MIAs.

### 2.6 CrEIN3 Regulates the Monoterpenoid Indole Alkaloids Biosynthesis by Binding to and Activating Enzymatic Gene Promoter

**FIGURE 5:**
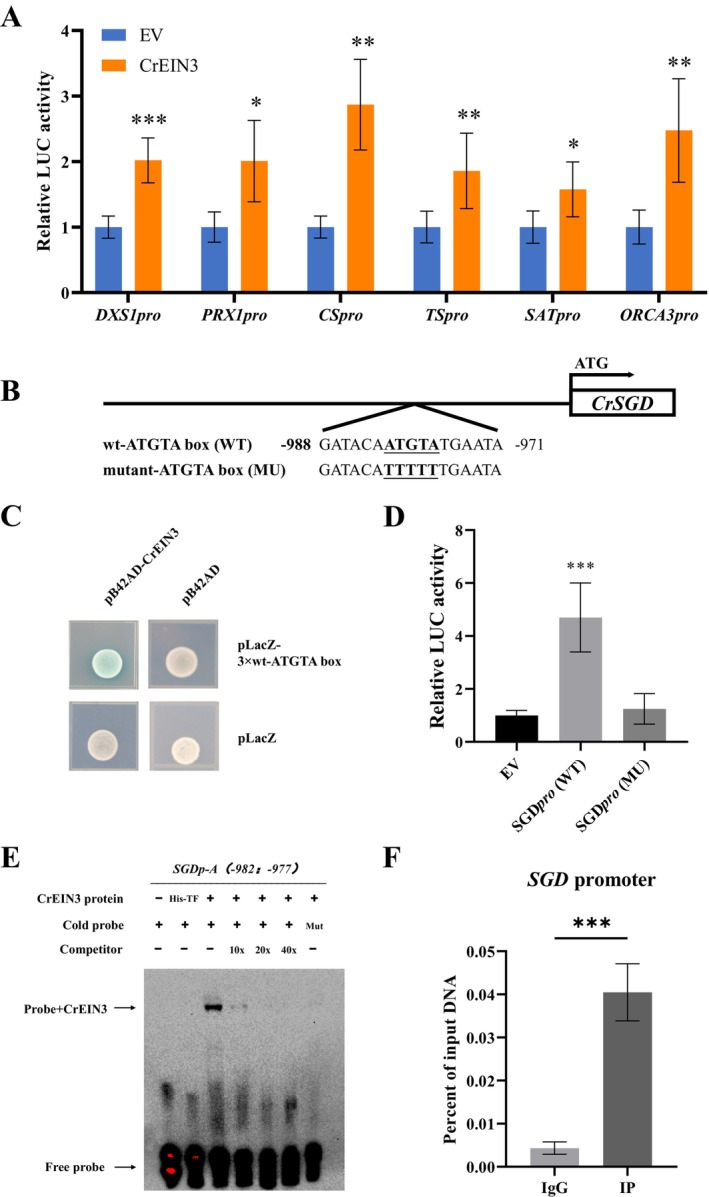
Identification of CrEIN3 as a key regulator modulating MIAs biosynthetic genes in *C. roseus* . (A) Transient Dual‐LUC assay of CrEIN3 with MIAs pathway genes (*PRX1, CS, TS, SAT* and *ORCA3*) promoters in tobacco. The LUC/REN values for the combination of empty effector construct p2300 and each reporter construct were set to 1. (B) Schematic diagrams of the SGD promoters, containing the potential CrEIN3 DNA binding sites (ATGTA‐box) numbered on the basis of their distance from the translational start site (ATG), which is set as +1. (C) CrEIN3 activates the *SGD* promoter as indicated by yeast one‐hybrid assays. EGY48A yeast expressing pB42AD or pB42AD‐CrEIN3 and ATGTA‐box fragments were grown on SD/−Ura/−Trp medium (20 mg l−1 X‐gal). The blue plaques show protein–DNA interactions. (D) Transient Dual‐LUC assay of CrEIN3 with the WT SGD promoter and the mutated SGD promoter in the ATGTA‐box. The LUC/REN values for the combination of empty effector construct and each reporter construct were set to 1. (E) EMSA showing CrEIN3 binds to ATGTA‐box in the *SGD* promoter. The His–CrEIN3 fusion protein and the labelled probe were used. Unlabeled probe was used as a cold competitor. 10×, 20× and 40× represent 10‐fold, 20‐fold and 40‐fold molar excess of unlabelled probe, respectively. His‐TF protein was included as a negative control. Mut, mutated probe. (F) ChIP‐qPCR analysis in CrEIN3‐GFP‐OE petals. *C. roseus* petals were inoculated with Agrobacterium carrying pHB‐CrEIN3‐GFP and isolated for input chromatin. GFP‐tagged CrEIN3‐chromatin complex was immunoprecipitated with an anti‐GFP antibody. A control reaction was processed at the same time using goat anti‐rabbit IgG. ChIP‐ and input‐DNA samples were quantified by qPCR using primers specific to the promoters of SGD gene. ChIP results are shown as percentages of input DNA. The error bars represent the means ± SD from three biological replicates, and Asterisks indicate statistically significant differences using Student's *t*‐test. *p < 0.05, **p < 0.01, ***p < 0.001.

To explore the regulatory impact and mechanism of CrEIN3 on MIAs biosynthetic genes, a dual‐LUC assay was conducted to assess the transactivation capability of CrEIN3. The results revealed that CrEIN3 was able to notably activate the promoters of *ORCA3, SGD, SAT, CS, TS* and *PRX1* (Figure 5A,D).

Y1H assays were conducted to investigate the direct binding of CrEIN3 to the promoters of enzyme genes. The Y1H analysis revealed that CrEIN3 bound to the SGD promoter region containing the ATGTA‐box, indicating that SGD is a direct target gene (Figure 5C). However, the promoters of the other genes (*ORCA3, SAT, CS, TS, PRX1*) showed negative results in Y1H (Figure S11). A dual‐LUC assay showed that CrEIN3 failed to activated the SGD promoter when its ATGTA‐box was mutated (Figure 5D). The results indicated that the ATGTA‐box is essential for CrEIN3 to activate and bind to the SGD promoter.

To validate this finding, an EMSA was conducted using the His‐CrEIN3 protein. This experiment confirmed that His‐CrEIN3 could indeed bind to the ATGTA‐box in the SGD promoter (Figure 5E). Subsequently, when the His‐CrEIN3 protein was replaced with His‐TF expressed using the pCold empty vector, no signal was detected (Figure 5E). These results suggest that CrEIN3 has the ability to independently and directly regulate MIA biosynthesis by binding to and activating the SGD promoter.

To further investigate the interaction between CrEIN3 and the *SGD* promoter in *C. roseus* plants, we performed chromatin immunoprecipitation‐quantitative PCR (ChIP‐qPCR) analysis. For this experiment, we used *C. roseus* petals transiently transformed with Agrobacterium containing pHB‐CrEIN3‐GFP plasmid, in which the expression of CrEIN3 fused with GFP at the C terminus was driven by a 35 s promoter. Immunoprecipitation with the anti‐GFP antibody greatly enriched the *SGD* promoter region containing the ATGTA‐box (spanning from −981 to −977 bp) (Figure 5B,F). In contrast, the IgG control antibody failed to enrich the SGD gene promoter. This result demonstrates that CrEIN3 directly binds to the promoter of *SGD* in vivo.

### 2.7 CrEIL1 Regulates the Monoterpenoid Indole Alkaloids Biosynthesis by Interacting With BIS2

Dual‐LUC assays showed that CrEIL1 significantly activated the promoters of *ORCA3, CrWRKY1, ASα, TDC, DXS1, LAMT, SLS1, STR, SGD, REDOX2, SAT* and *DAT* compared to the control (Figure S12), indicating that CrEIL1 positively regulates MIA biosynthesis. However, Y1H analysis revealed that CrEIL1 does not bind directly to these promoters, suggesting it may regulate these MIA genes indirectly, possibly via interactions with other transcription factors.

Co‐expression network analysis revealed a strong correlation (with a correlation coefficient > 0.95) between CrEIL1 and BIS2, as well as with several other TFs (Figure S13). To identify CrEIL1‐interacting partners, we performed a yeast two‐hybrid (Y2H) screen. Full‐length coding sequences of candidate proteins, including BIS2, were cloned into the GAL4 DNA‐binding domain (BD) vector as baits, whereas the full‐length CrEIL1 sequence was fused to the GAL4 DNA‐activation domain (AD). The Y2H assay showed that CrEIL1 specifically interacts with BIS2 among the tested TFs (Figure S14).

**FIGURE 6:**
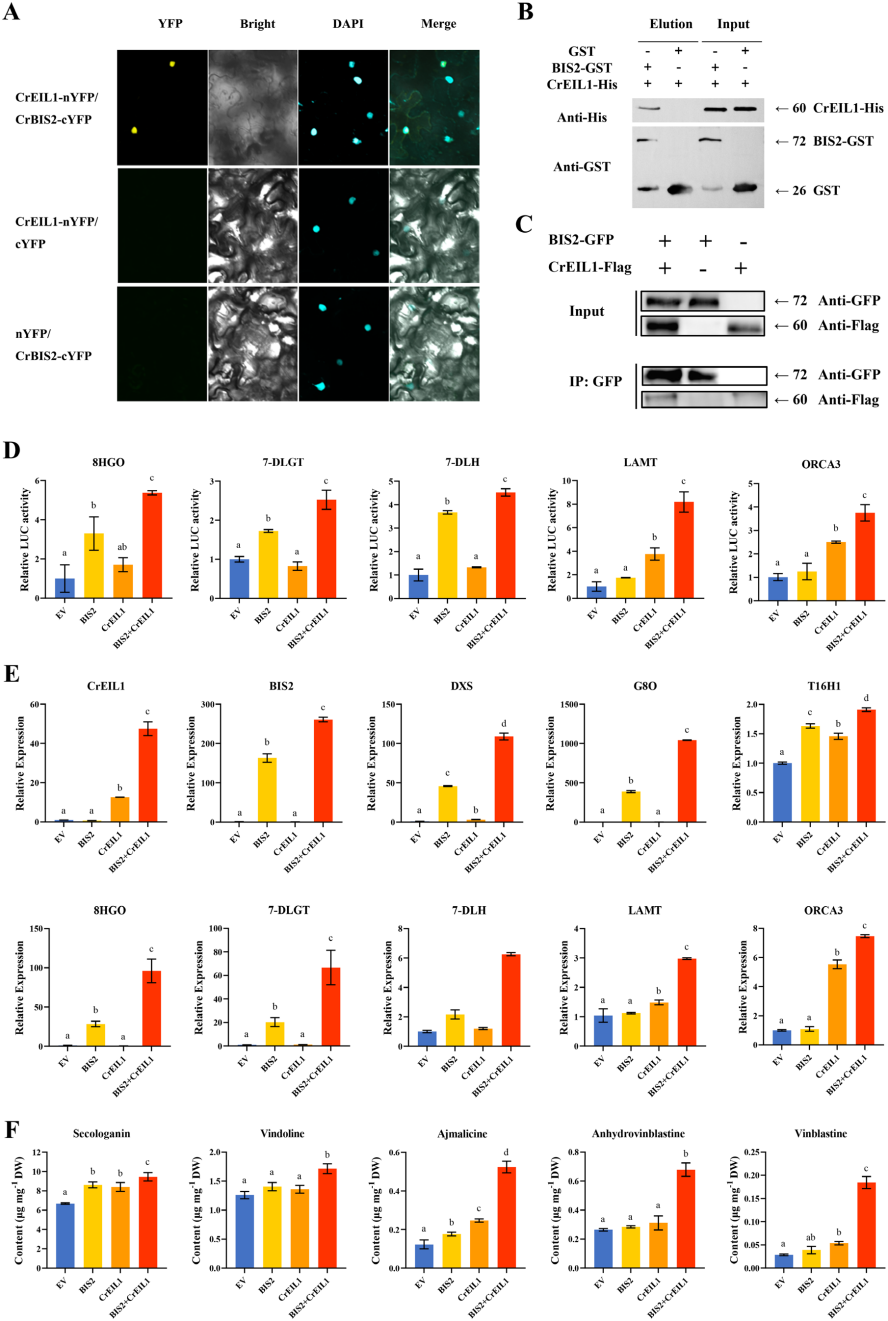
Interaction between CrEIL1 and BIS2 and their synergistical effect on the MIA biosynthesis. (A) Bimolecular fluorescence complementation (BiFC) analysis of the interaction between CrEIL1 and CrBIS2 in *N. benthamiana* cells. The N‐terminal fragment of YFP was fused to the N‐terminus of CrEIL1 (nYFP–CrEIL1), and CrBIS2 was fused to the C‐terminal fragment of YFP (CrBIS2–cYFP). Three independent transfection experiments were performed. Scale bar = 50 μm. (B) In vitro pulldown assays of CrEIL1 and BIS2 recombinant proteins. CrEIL1‐His proteins were pulled down with BIS2‐ GST and further detected on Western blots probed with anti‐His antibody. Experiments were carried out three times, and representative results are shown. (C) Co‐immunoprecipitation studies of CrEIL1 and BIS2 interaction in *N. benthamiana* leaves. Total protein extracts from *N. benthamiana* leaves infiltrated with constructs harbouring BIS2‐GFP and CrEIL1‐FLAG were immunoprecipitated with anti‐FLAG antibody. The co‐immunoprecipitated proteins were detected by anti‐GFP antibody. Experiments were repeated three times and similar results were obtained. (D) Activation of the *8‐HGO, 7‐DLGT, 7‐DLH, LAMT* and *ORCA3* promoters by CrEIL1 and BIS2 proteins in *N. benthamiana* leaves. The LUC/REN values for the combination of empty effector construct p2300 and each reporter construct were set to 1. Three independent transfection experiments were performed. The reporter strain harbouring *8‐HGO/7‐DLGT/7‐DLH/LAMT/ORCA3pro:LUC* was mixed with the effector strains harbouring 35Spro:CrEIL1 and 35Spro:BIS2 at a ratio of 1:1:1. The data represent the means ± SD of three replicates from three independent experiments. a, b, c, d: Significant difference (*p* < 0.05), one way ANOVA. (E) Expression levels of *CrEIL1, BIS2, DXS, G8O, T16H1, 8‐HGO, 7‐DLGT, 7‐DLH, LAMT* and *ORCA3* in different *C. roseus* petal samples including *CrEIL1‐BIS2* co‐overexpression (BIS2‐CrEIL1), *CrEIL1* overexpression, *BIS2* overexpression and petals transformed with the empty vector. *N2227* was used as the internal standard. The petals transformed with the empty vector served as controls. The data represent the means ± SD of three replicates from three cutting propagations. a, b, c, d: Significant difference (*p* < 0.05), one way ANOVA. (F) LC–MS analysis of MIAs in the petals of different *C. roseus* plants including *CrEIL1‐BIS2* co‐overexpression (BIS2‐ CrEIL1), *CrEIL1* overexpression, *BIS2* overexpression and petals transformed with the empty vector. The data represent the means ± SD of three replicates from three cutting propagations. a, b, c, d: Significant difference (*p* < 0.05), one way ANOVA.

Bimolecular fluorescence complementation (BiFc) assays in *Nicotiana benthamiana* confirmed the direct interaction between CrEIL1 and BIS2 (Figure 6A). Pull‐down assays further supported this interaction (Figure 6B), and coimmunoprecipitation (Co‐IP) assays showed that CrEIL1‐flag was specifically coimmunoprecipitated with BIS2‐GFP (Figure 6C). Collectively, these results demonstrate a consistent interaction between CrEIL1 and BIS2 both in vitro and in vivo.

### 2.8 Interaction Between CrEIL1 and BIS2 Enhances the Monoterpenoid Indole Alkaloids Biosynthesis

To determine how the CrEIL1–BIS2 interaction influences the MIA biosynthetic pathway, we first used dual‐LUC assays in *N. benthamiana* to test whether their transcriptional activities are altered. Expressed alone, BIS2 activated the *8‐HGO, 7‐DLGT* and *7‐DLH* promoters, whereas CrEIL1 specifically activated the *LAMT* and *ORCA3* promoters. Co‐expression of BIS2 and CrEIL1, however, significantly enhanced activation of all five promoters (*8‐HGO, 7‐DLGT, 7‐DLH, LAMT* and *ORCA3*) (Figure 6D), indicating that their physical interaction synergistically boosts their transcriptional activities.

Additionally, we co‐overexpressed *CrEIL1* and *BIS2* in *C. roseus* petals. Compared with lines overexpressing one gene alone, the co‐overexpression lines exhibited significantly higher transcript levels of *DXS, G8O, 8‐HGO, 7‐DLGT, 7‐DLH, LAMT, T16H1* and *ORCA3* (Figure 6E). This transcriptional up‐regulation corresponded to increased accumulation of secologanin, vindoline, ajmalicine, anhydrovinblastine and vinblastine (Figure 6F). Specifically, anhydrovinblastine and vinblastine contents in the co‐overexpression lines rose 1.2‐ and 2.4‐fold relative to the CrEIL1‐only lines, and 1.6‐ and 5.5‐fold relative to the control, respectively. Ajmalicine increased 1.1‐fold versus the CrEIL1‐only lines and 3.3‐fold over the control. These results demonstrate that the CrEIL1–BIS2 interaction synergistically activates iridoid‐pathway genes and enhances downstream MIA production.

## 3 Discussion

The EIN3/EILs proteins are the key ethylene‐signalling regulators and contribute to the intricate network of primary and secondary metabolic pathways of plants. Ethylene can induce the accumulation of MIAs. However, the specific ethylene‐inducible transcription factors (TFs) responsible for MIA biosynthesis have not been identified. In our study, we conducted a comprehensive analysis of the MIAs‐related metabolome and transcriptome under ethylene or JA treatment, which allowed us to establish ethylene and JA regulatory networks associated with MIA accumulations. Our integrated metabolomic and transcriptomic analysis revealed that ethylene and JA signals both promote MIA biosynthesis, but with distinct regulatory features and time courses. Metabolite profiling (Figure 1) indicated that ethylene treatment led to a more sustained and significant accumulation of late‐stage MIAs such as anhydrovinblastine and catharanthine, with a particularly pronounced increase in anhydrovinblastine (65.27%) at 24 h post‐treatment. In contrast, MJ treatment caused a rapid but transient rise in vindoline and tryptamine at 3 h, but less effect on bisindole alkaloids, consistent with previous findings that ethylene is more effective in promoting late‐pathway MIA production (Chen et al. 2017; Wang et al. 2016).

Based on these findings, we linked gene expression modules to MIA accumulations at 0 h, 3 h and 24 h under ETH and MJ treatments and, using GENIE3, constructed (i) a regulatory network for anhydrovinblastine and catharanthine associated with the green and pink modules and (ii) a network for vindoline linked to the turquoise module. This analysis led to the discovery and functional characterisation of two novel EIN3/EIL TFs, CrEIN3 and CrEIL1, that act as key mediators of ethylene signalling in *C. roseus* .

In *Arabidopsis*, EIN3 and EIL1 are critical TFs that regulate most ethylene‐responsive phenotypes (Chao et al. 1997; Alonso et al. 2003; An et al. 2018). The ethylene signal activates EIN3 and EIL1 by increasing their protein stability (Zhao et al. 2020). Furthermore, EIN3/EILs are involved in regulating the biosynthesis of various plant secondary metabolites, including tocopherol in *Arabidopsis thaliana* , natural rubber in *Hevea brasiliensis* , carotenoid in papaya, and anthocyanin in apple (Cela et al. 2009; Yang et al. 2015; Fu et al. 2017; An et al. 2018). Based on the transcriptomic and metabolomic study, we identified *CrEIN3* in the pink module and *CrEIL1* in the turquoise module using GENIE3 to predict the gene regulatory network. We further demonstrated that ethylene‐induced MIA accumulations is dependent on CrEIN3 and CrEIL1 (Figure 3).

Previous studies have shown that ORCAs, CrMYC2 and BIS1/2 upregulate genes in the indole or iridoid pathways, while ORCA3 activates STR expression by binding to the promoter (Van Der Fits and Memelink 2000; Zhang et al. 2011; Van Moerkercke et al. 2015). The genes involved in the steps from strictosidine to catharanthine or tabersonine are not under the control of JA‐inducible TFs, such as SGD. The engineered de‐repressed CrMYC2a was reported to activate SGD (Schweizer et al. 2018), while its transcriptional regulation remains unknown in *C. roseus* . Our results of overexpression and VIGS experiments demonstrated that CrEIN3 upregulates the transcripts of MIA activators, such as *ORCA3* and *CrERF5*, while downregulating the transcripts of MIA repressors, such as *ZCT1* and *GBF1* (Li et al. 2015). In addition, Dual LUC assays revealed that CrEIN3 activates the transcription activity of *ORCA3, SGD, CS, TS, REDOX2, SAT* and *PRX1*. Y1H, EMSA and ChIP‐qPCR experiments demonstrated that CrEIN3 directly modulates the ethylene signal and regulates MIA production by binding to the *SGD* promoter in *C. roseus* , and CrEIN3 activation on the SGD promoter was dependent on the ATGTA‐box (Figure 5E). CrEIN3 enhances the expression of MIA enzymatic genes, including *SGD, CS, TS* and *PRX1*, thereby increasing the metabolic flux towards downstream MIAs, such as vinblastine and anhydrovinblastine.

Interestingly, CrEIL1 exhibits a distinct mode of regulating MIA biosynthesis. Both overexpression and silencing of CrEIL1 resulted in similar effects as CrEIN3 on the expression of MIA activators and repressors. Additionally, CrEIL1 showed a stimulatory effect on iridoid pathway genes and significantly upregulated the secologanin accumulation. Dual LUC assays further confirmed that CrEIL1 activated the promoters of iridoid pathway genes, such as *DXS1, LAMT* and *SLS*. However, no direct binding of CrEIL1 to these promoters was observed, suggesting the involvement of putative interacting proteins that assist CrEIL1 in its activation role. Our datasets revealed a strong correlation between *CrEIL1* and *BIS2* expression. BIS2 is a JA‐responsive bHLH TF known to regulate iridoid pathways as BIS1 in *C. roseus* (Van Moerkercke et al. 2016). We demonstrated the interaction between CrEIL1 and BIS2 in vitro and in vivo (Figure 6). Western blot and fluorescence microscopy analyses of CrEIL1 and BIS2‐GFP co‐expressed in *C. roseus* petals and tobacco leaves showed that the interaction with CrEIL1 does not alter BIS2 protein abundance or nucleus localization (Figures S15 and S16).

Ethylene and JA are two major plant hormones that synergistically regulate plant development and confer tolerance to biotic stress (Zhu et al. 2011). In *Arabidopsis*, EIN3 interact with MYC2 to repress both genes, and cause the antagonism between JA and ethylene during apical hook development (Song et al. 2014; Zhang et al. 2014). In *C. roseus* , CrEIL1 was found to interact with BIS2 and their interaction enhanced the transcriptional activation of both CrEIL1 and BIS2, and significantly upregulated the expression levels of iridoid pathway genes (*G8O, 8‐HGO, 7‐DLGT, 7‐DLH*), as well as the *LAMT* and *ORCA3* genes. The enzyme of G8O in iridoid pathway was known as a key enzymic limiting step to MIA pathway (Van Der Fits and Memelink 2000). This synergistic effect led to a marked increase in the accumulation of secologanin, vindoline, ajmalicine, anhydrovinblastine and vinblastine. These findings suggest that ethylene and JA might have the synergism on upregulating the MIA biosynthesis, which is worth further investigation in future. The interaction between CrEIL1 and BIS2 makes the co‐expression of both genes a more potent strategy to enhance the MIA biosynthesis pathway compared to the expression of a single gene alone.

**FIGURE 7:**
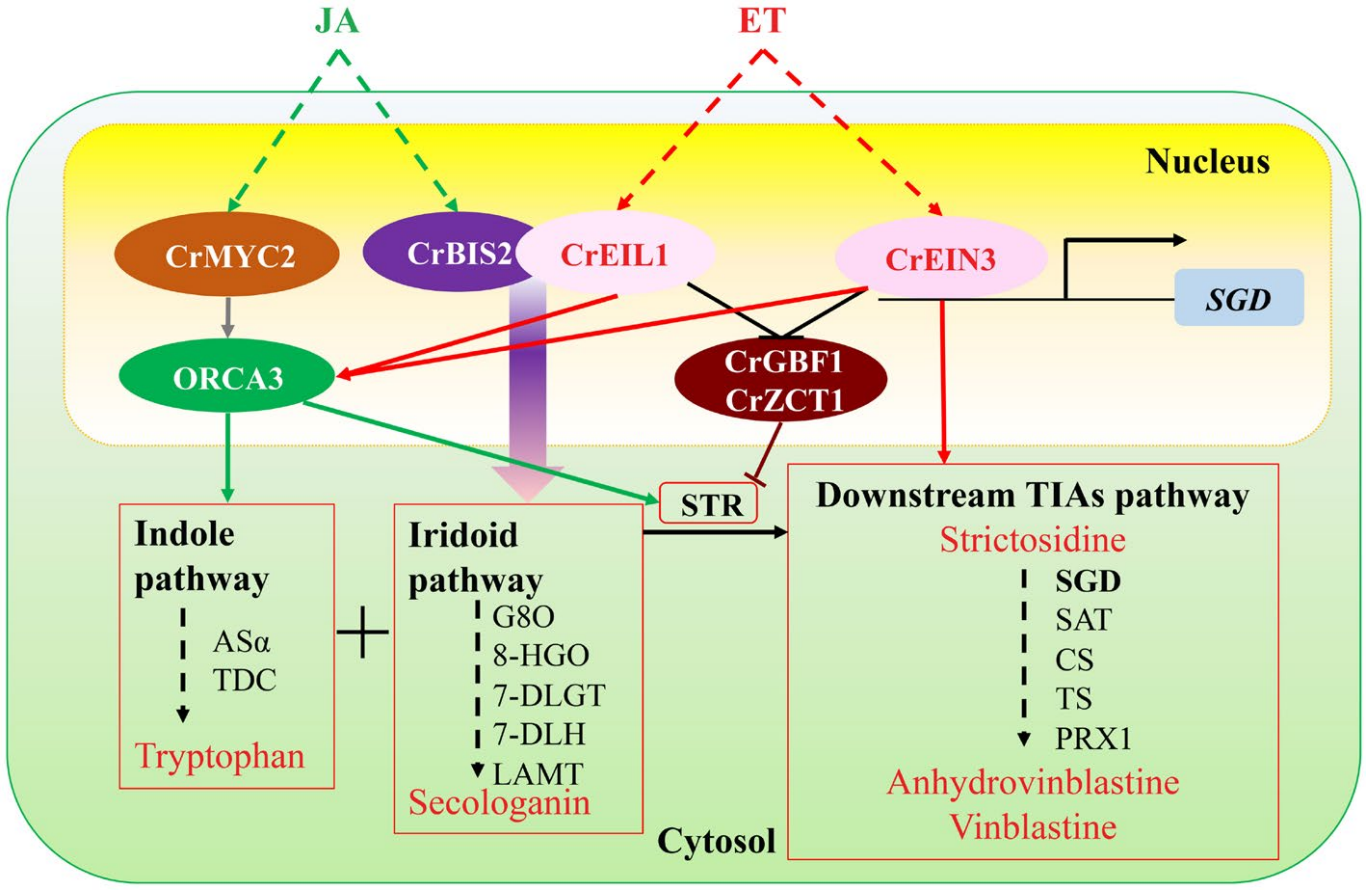
ET‐induced regulatory model consisting of CrEIN3 and CrEIL1 in the regulation of the MIAs production in *C. roseus* . Under the induction of ET signal, CrEIN3 activated the downstream MIAs pathway by binding to the *SGD* promoter, and also upregulated the expression of *SAT*, *CS*, *TS* and *PRX1*. CrEIL1 interacted with BIS2 and strengthen the activation on the transcriptional activity of iridoid pathway genes. Meanwhile, both CrEIN3 and CrEIL1 activated the expression of *ORCA3* and repressed the expression of *CrGBF1* and *CrZCT1* to enhance the upstream MIAs metabolism, which further lead to a boost of vinblastine accumulation.

Moreover, the dramatic loss of ET‐induced catharanthine and anhydrovinblastine accumulation in VIGS‐*CrEIN3*‐*CrEIL1* double‐silenced plants (Figure 3A,B) provides compelling genetic evidence that both TFs are indispensable and likely major conduits for the ET signal in regulating MIA biosynthesis, particularly the bisindole pathway. This functional redundancy or cooperation ensures robust pathway activation. Our study reveals a sophisticated bifurcated regulatory model for ET action in *C. roseus* : (1) CrEIN3 directly targets and activates the gateway enzyme gene SGD, propelling the downstream MIA pathway; (2) CrEIL1, via interaction with BIS2, potently enhances the upstream iridoid pathway. Simultaneously, both TFs converge on amplifying the ORCA3 activator module and repressing the GBF1/ZCT1 repressor module, creating a multi‐layered regulatory network that maximises MIA flux towards vinblastine and related alkaloids (Figure 7). Evaluating the functionality and impact of CrEIN3/CrEIL1 and key regulated enzymes (e.g., SGD) in heterologous systems such as yeast or engineered plant hosts represents an important future step towards assessing their biotechnological utility for MIA production.

## 4 Conclusion

Our research offers new insights into the role of ethylene signalling in MIA biosynthesis, highlighting the involvement of key components, CrEIN3 and CrEIL1. Collectively, our results indicate that ethylene enhances MIA biosynthesis in *C. roseus* through the regulatory model consisting of two key regulators: (i) CrEIN3 directly increases the transcriptional activation of the *SGD* promoter; and (ii) the interaction between CrEIL1 and BIS2 facilitates the iridoid pathway, further promoting MIA biosynthesis to bisindole alkaloids, like anhydrovinblastine and vinblastine (Figure 7).

## 5 Methods

### 5.1 Plant Materials

Seeds of *Catharanthus roseus* (cv. Vitae Rose Red) were purchased from Floranova Ltd. *Nicotiana benthamiana* plants were cultivated in the greenhouse under a 16‐h light/8‐h dark cycle per day. Seeds of *Arabidopsis ein3‐1 eil1‐3* mutant were from Tsinghua‐Peking Center for Life Sciences. The mutant *ein3‐1 eil1‐3* was previously described (Song et al. 2014). *Arabidopsis* seeds were sterilised with 20% bleach, plated on Murashige and Skoog (MS) medium, chilled at 4°C for 3 days, and then transferred to a growth room at 25°C under a 16‐h/8‐h light/dark photoperiod.

### 5.2 Plant Transformation

The plasmids p2300‐*35S:CrEIN3* and p2300‐*35S:CrEIL1* were generated, respectively. The GV3101 strains of *Agrobacterium tumefaciens* containing above constructs individually were used to transform *ein3 eil1* mutant with in planta vacuum infiltration method (Bechtold et al. 1993). Kanamycin‐resistant T1, T2 plants were selected by planting seeds on MS medium supplemented with 1% sucrose and 50 μg/mL kanamycin and transferred to soil. T2 plants with kanamycin resistance were used for hook phenotype analysis as described previously (Song et al. 2014).

### 5.3 Hormone Treatments

For the plant hormonal treatments, 4‐week‐old *C. roseus* seedlings were subjected to foliar spraying with either 100 μM ethephon (ETH) or 100 μM methyl jasmonate (MJ). Samples were collected at 0, 3, 6, 24 and 72 h post‐treatment for targeted MIAs analysis. Specifically, samples collected at 0, 3 and 24 h were used for metabolomic analysis and RNA‐seq. For the mock treatment, seedlings were sprayed with ddH_2_O. The experiments were replicated three times to validate the findings.

### 5.4 NMR Measurements and Metabolomic Analysis

*C. roseus* leaves samples were prepared and extracted for NMR measurements as described previously (Kim, Choi, and Verpoorte 2010; Pan et al. 2014). The ^1^H‐NMR spectra were recorded using a Bruker DMX 600 spectrometer (Bruker, Karlsruhe, Germany). The parameters for two‐dimensional NMR spectra such as *J*‐resolved, correlated spectroscopy (COSY) (Kim, Saifullah, et al. 2010).

^1^H‐NMR spectra were automatically binned using AMIX software (Version3.7, Biospin, Bruker). Spectral intensities were scaled to total intensity and the region of *δ* 0.30–*δ* 10.00 was reduced to integrated regions of 0.04 ppm each as previous study (Pan et al. 2014). SIMCA‐*P* ^+^ software (Version 13.0, Umetrics, Umeå, Sweden) was used for principal component analysis (PCA) and partial least‐square (PLS) modelling with Pareto‐scaled data of binned ^1^H‐NMR.

### 5.5 RNA Sequencing and Data Analysis

The cDNA libraries were constructed for each RNA sample using the TruSeq Stranded mRNA Library Prep Kit (Illumina Inc.) according to the manufacturer's instructions. Then the cDNA libraries were used for 150 bp paired‐end sequencing on Illumina HiSeq XTen platform. Before read mapping, clean reads were obtained from the raw reads by removing the adaptor sequences and low‐quality reads. The clean reads were then aligned to *Catharanthus roseus* genome (GCA_000949345.1, NCBI and Data for: Single‐cell multi‐omics in the medicinal plant *Catharanthus roseus* , DRYAD, https://doi.org/10.5061/dryad.d2547d851) using the Hisat2 (Kim et al. 2015). HTseq was used to get gene counts and FPKM method was used to determine the gene expression (Anders et al. 2015). Hierarchical cluster (HCL) analysis was performed using a Multi Experiment Viewer (MEV, v.4.9.0) as previous study (Pan et al. 2019). The differential gene expression analysis and weighted gene co‐expression network analysis (WGCNA) were performed as described in details (Figure S4).

Based on module correlation and module functional annotation, the module associated with the metadata annotation of the cluster was selected. R package GENIE3 was then employed to predict the regulatory relationships between genes within the module (Zheng et al. 2024). To construct the gene regulatory network, only gene pairs with a weight value greater than or equal to 0.006 were considered. The constructed gene regulatory networks were imported into Cytoscape software for visualisation and analysis.

### 5.6 Subcellular Localization

The full‐length ORF of *CrEIN3* and *CrEIL1* without the stop codon was amplified by PCR from *C. roseus* leaf cDNA library with primers and inserted into BamHI and SpeI sites of the pHB‐*GFP* expression vector to generate the pHB‐*CrEIN3‐GFP* and pHB‐*CrEIL1‐GFP*, pHB‐*BIS2‐GFP* constructs. pHB‐*CrEIN3‐GFP*, pHB‐*CrEIL1‐GFP* and pHB‐*BIS2‐GFP*, pHB‐*GFP* were then introduced into *Agrobacterium tumefaciens* strain GV3101 for *N. benthamiana* leaf transient expression. After 24 h incubation in darkness and 24 h incubation in low‐light conditions, GFP signals were observed by a confocal laser microscopy.

### 5.7 RNA Isolation and Real‐Time Quantitative PCR

To assess the transcript levels of candidate genes in various tissues and hormone‐treated seedlings, tissue samples including young leaves, roots, stems, flowers, seed pods and old leaves were collected. Total RNA from all samples was extracted using the SuperReal PreMix PLUS kit (Tiangen Biotech, Beijing, China), and cDNA synthesis was performed using the PrimeScript RT reagent kit (Takara, Shiga, Japan). Transcript levels were quantified using the comparative cycle threshold (Ct) method. The primers utilised for real‐time quantitative PCR (RT‐qPCR) can be found in Table S1.

### 5.8 Transient Transformation in *Catharanthus roseus* Petals

Flower petals of 1‐year‐old *C. roseus* plants (grown under greenhouse conditions) were infiltrated with *A. tumefaciens* GV3101 harbouring the respective constructs pHB‐*CrEIN3‐GFP*, pHB‐*CrEIL1‐GFP* and pHB‐*GFP* as an empty vector (EV) for overexpression as previously described (Schweizer et al. 2018). After 48 h of incubation with *A. tumefaciens* solution, petals from five different flowers per sample were cut in two and immediately flash‐frozen in liquid nitrogen. One sample was used for RNA extraction for gene expression analysis and the other sample was used for metabolite profiling. Three replicates were made for each sample.

### 5.9 Virus‐Induced Gene Silencing

Seedlings of *C. roseus* with two to three pairs of true leaves were used for virus‐induced gene silencing (VIGS). The genomic DNA of *C. roseus* was used as template to amplify the partial fragment of *CrEIN3* (362 bp) and *CrEIL1* (351 bp) with specific primers (Table S1). The PCR‐generated fragment was then introduced into the terminal vector pTRV2 by Gateway LR ClonaseTM II (Invitrogen, Thermo Fisher Scientific, United States). The purified plasmid pTRV2‐*CrEIN3*, pTRV2‐*CrEIL1*, pTRV2‐EV were respectively transformed into *A. tumefaciens* strain GV3101 for VIGS as described previously (Pan et al. 2019). For CrEIN3 and CrEIL1 both‐VIGS line, *A. tumefaciens* cultures harbouring pTRV2‐CrEIN3/pTRV1 or pTRV2‐CrEIL1/pTRV1 were mixed in equal volume to inject into plants. Each treatment was injected into 18 plants. After injection, the plants were cultured in a constant temperature incubator, and harvested or treated with 100 μM ETH 3 weeks after inoculation. After recording the fresh weights of harvested materials, the samples were frozen in liquid nitrogen. Three replicates were made for each sample.

### 5.10 Metabolite Extraction and Analysis by UPLC‐Q/TOF MS

For alkaloid extraction, pedals samples were flash frozen in liquid Nitrogen. Samples were extracted with 1 mL methanol and fully mixed with shaker. After sonicated for 45 min, samples were centrifuged for 10 min at 12,000 rpm. Then, the supernatants were gently removed to new tube for next analysis. UPLC‐Q/TOF MS analyses were performed as described previously (Pan et al. 2019). Data acquisition, handling and instrument control were performed using MassLynx 4.1 software. Samples were applied in triplicate for quantification using calibration curves of the standards (Table S3; Figure S8).

### 5.11 Dual‐Luciferase Assays

Dual‐luciferase assays (dual‐LUC) followed the protocol described in a previous study (Shan et al. 2014). Plasmids p2300‐CrEIN3, p2300‐CrEIL1 and p2300‐BIS2 were introduced into *Agrobacterium tumefaciens* strain GV3101 for their role as effectors. The promoter constructs pGREEN‐DXS‐pro, pGREEN‐TDC‐pro, pGREEN‐ASα‐pro, pGREEN‐STR‐pro, pGREEN‐SGD‐pro, pGREEN‐CS‐pro, pGREEN‐TS‐pro, pGREEN‐REDOX2‐pro, pGREEN‐SAT‐pro, pGREEN‐DAT‐pro, pGREEN‐PRX1‐pro, pGREEN‐CrWRKY1‐pro, pGREEN‐ORCA3‐pro, pGREEN‐8HGO‐pro, pGREEN‐7‐DLGT‐pro, pGREEN‐7‐DLH‐pro, pGREEN‐LAMT‐pro and pGREEN‐SLS‐pro were individually linked to the luciferase reporter gene and co‐transformed with the helper plasmid pSoup19 into GV3101. To generate mutant SGD promoter, ATGTA‐box motif in the SGD promoter were changed to (TTTTT) by site‐directed mutagenesis using the protocol described previously (García‐Nafría et al. 2016). The empty vector P2300 (EV) served as the negative control. The 35S promoter‐driven Renilla luciferase was employed as an internal reference. Following 48 h of growth under low‐light conditions, leaf samples were harvested for dual‐LUC assays using commercial dual‐LUC reaction reagents (Promega). Each sample was subjected to six biological replicates.

### 5.12 Yeast One‐Hybrid Assay

In the yeast one‐hybrid assay (Y1H), the full‐length coding sequences of *CrEIN3* were inserted into the pB42AD vector to create the plasmid pB42AD‐*CrEIN3*. Triple tandem copies of the p*SGD* wt‐ATGTA‐box motif (GATACAATGTATGAATA) and the mutant‐ATGTA‐box motif (GATACAtttttTGAATA) were integrated into the reporter plasmid pLacZ between EcoRI and XhoI sites. The empty pB42AD and pLacZ vectors were utilised as negative controls. Various combinations were co‐transformed into the yeast strain EGY48a, and the transformants were grown on SD/−Trp/−Ura medium and assessed on SD/−Trp/−Ura medium supplemented with X‐gal.

### 5.13 Electrophoretic Mobility Shift Assays

For the electrophoretic mobility shift assays (EMSA) assay, the pCold‐*CrEIN3* vector was constructed and introduced into *Escherichia coli* strain Rosetta (DE3) (TransGen Biotech, China). Expression of the His‐tagged fusion protein was induced by adding 200 μM isopropyl‐D^−1^‐thiogalactopyranoside (IPTG) and incubating at 16°C for 14 h, followed by purification using nitrilotriacetic acid (Ni‐NTA) agarose (Invitrogen Life Technologies). EMSA assays were conducted using the LightShift Chemiluminescent EMSA Kit (Thermo, USA) according to the manufacturer's instructions (Thermo Fisher Scientific, Waltham, MA, USA). The ATGTA and ATGTA‐mutant DNA probes were synthesised by Sangon (Shanghai, China). The primers used in the EMSA are detailed in Table S1.

### 5.14 ChIP‐qPCR Analysis

*C. roseus* petals infiltrated with Agrobacterium GV3101 carrying pHB‐*CrEIN3‐GFP* were used for ChIP qPCR analysis. Briefly, chromatin complex was isolated from 0.8 g fixed and frozen tissue and was sonicated for 60 min (30‐s on and 30‐s off cycles) with a Bioruptor JY88‐II (Ningbo Xinyi Ultrasonic Equipment Co. Ltd). Immunoprecipitation of chromatin complex was done using Anti‐GFP mAb‐Magnetic beads (Abcam) or Goat Anti‐Rabbit IgG magnetic beads (Proteintech) as a negative control. The immunoprecipitated DNA was purified using the Wizard SV Gel and a PCR Clean‐Up system (Cell Signalling Technology, CST), and the purified DNA was used for qPCR analysis. Table S1 lists the primers used for qPCR.

### 5.15 Yeast‐Two‐Hybrid

For the yeast‐two‐hybrid assay (Y2H), the open reading frame (ORF) of *BIS2* was individually inserted into the pGBKT7 vector as the bait, while the ORF of *CrEIL1* was cloned into the pGADT7 vector as the prey. Equal quantities of the AD and corresponding BD constructs were introduced into the yeast strain AH109 using the Matchmaker GAL4 yeast two‐hybrid system. The transformed yeast cells were cultured on selective media, including SD/Leu−/Trp−/His−, SD/Leu−/Trp−/His−/Ade− and SD/Leu−/Trp−/His−/Ade−/3‐amino‐1,2,4‐triazole (3‐AT). The plates were then incubated for 3 days at 30°C.

### 5.16 Bimolecular Fluorescence Complementation Assay

For the bimolecular fluorescence complementation (BiFC) assays, cDNA fragments encoding BIS2 were cloned and fused with the N‐terminus of yellow fluorescent protein (nYFP) to generate pxy104–BIS2, while cDNA fragments encoding CrEIL1 were fused with the C‐terminus of YFP (cYFP) to create pxy106–*CrEIL1*. The constructed plasmids were transformed into *Agrobacterium tumefaciens* strain GV3101. The transformed GV3101 strains were then infiltrated into the young leaves of 4‐week‐old *Nicotiana benthamiana* plants. YFP signals were visualised using confocal laser microscopy (Leica TCS SP5‐II).

### 5.17 Pull‐Down Assays

The full‐length coding sequence of CrEIL1 was inserted into the pCold vector and transformed into *Escherichia coli* strain Rosetta (DE3) to generate the His‐CrEIL1 protein for pull‐down assays. The *Escherichia coli* strain Rosetta (DE3) carrying the pGEX4T‐BIS2 plasmid (GE Healthcare) produced the GST‐BIS2 protein, as previously described (Ma et al. 2018). Each experiment was performed in triplicate.

### 5.18 Co‐Immunoprecipitation Assays

To perform co‐immunoprecipitation (Co‐IP) assays, the full‐length coding region of *CrEIL1* was inserted into the pHB‐FLAG vector, while the open reading frame of BIS2 was cloned into the pHB‐GFP vector. The recombinant plasmids and empty vectors (EVs) were transformed into GV3101. The plasmid‐carrying strains were mixed and injected into *N. benthamiana* leaves. After 48 h of incubation, the leaves were collected and ground to a powder using liquid nitrogen. Co‐IP assays were then conducted, as previously described (Ma et al. 2018). Briefly, for each sample, 10 μL of rabbit anti‐GFP antibody and 15 μL of Protein G Sepharose (both from GE Healthcare, Bucks, UK) were incubated for 2 h at 4°C. The total proteins from tobacco were added to the Protein G Sepharose and incubated for an additional 2 h at 4°C. The proteins were detected using anti‐GFP (AbMart, Shanghai, China) and anti‐Flag antibody (Sigma‐Aldrich).

### 5.19 Data Statistical Analysis

All experiments were conducted with three replicates. Statistical analysis was performed using the student's t‐test or one way ANOVA by SPSS (version 14.0, Chicago, IL, USA). The values are mean ± SD for replicates in each group. *p* values ≤ 0.05 were considered as significant.

## Author Contributions

Q.P. planned and designed the research. B.D., Q.M., C.O., Y.P. and H.L. performed experiments. B.D., Q.M., C.O., X.F., L.L. and Y.W. analyzed data. B.D. and Q.M. wrote the manuscript. K.T. and Q.P. revised the manuscript and helped improve the manuscript.

## Conflicts of Interest

The authors declare no conflicts of interest.

## Supporting information


**Figure S1:** NMR spectrum of *C. roseus* plants.
**Figure S2:** Differential gene expression analysis.
**Figure S3:** qPCR and HCL analysis of key genes in TIA biosynthetic pathway under MJ and ETH treatment.
**Figure S4:** WGCNA analysis and co‐expression network.
**Figure S5:** Complementation of Arabidopsis *ein3eil1* mutantion by *CrEIN3* and *CrEIL1* genes.
**Figure S6:** Silencing efficiency of *CrEIN3* and *CrEIL1* via VIGS.
**Figure S7:** Phylogenetic analysis of CrERF5 and other known EIN3/EIL TFs from ten plant species.
**Figure S8:** The calibration curves, linear regression equation and correlation coefficient (R^2^) of tryptamine, secologanin, ajmalicine, catharanthine, vindoline, anhydrovinblastine and vinblastine.
**Figure S9:** Western blotting results of CrEIL1‐GFP overexpressed petals and CrEIN3‐GFP overexpressed petals.
**Figure S10:** Regulatory effect of CrEIL1 on TIAs biosynthesis in *C. roseus* .
**Figure S11:** Yeast one‐hybrid assays of CrEIN3 with the *ORCA3, SAT, CS, TS* and *PRX1* promoters.
**Figure S12:** Transient Dual‐luciferase assay of CrEIL1 with TIAs pathway genes promoters in tobacco.
**Figure S13:** Co‐expression network for *CrEIL1*.
**Figure S14:** Yeast two‐hybrid (Y2H) confirmed that CrEIL3 interacted with BIS2.
**Figure S15:** Western blotting results of BIS2‐GFP overexpressed petals and BIS2‐GFP/CrEIL1 co‐overexpressed petals.
**Figure S16:** Subcellular localization of BIS2‐GFP and BIS2‐GFP/CrEIL1 in tobacco leaves.
**Table S1:** List of primers used in this work.
**Table S2:** ^1^H NMR chemical shifts (δ) and coupling constants (Hz) of identified metabolites based on ^1^H‐NMR, *J*‐resolve, COSY, HSQC and references.
**Table S3:** MIAs and precursors identified in *Catharanthus roseus* by UPLC‐Q/TOF MS.
**Table S4:** Statistical analysis for selected signals from the NMR spectrum of ethylene treated, MeJA treated and control samples.

**Dataset: S1.** Untargeted and targeted metabolites content.

**Dataset: S2.** Summary of transcriptome mapping under ETH and MJ treatments in *Catharanthus roseus* .

**Dataset: S3.** Gene expression of *C. roseus* under ETH and MJ treatments.

**Dataset: S4.** Summary of co‐expression gene modules.

**Dataset: S5.** Trait modules correlation.

**Dataset: S6.** The coexpression network of catharanthine and anhydrovinblastine metabolism under ETH or MJ treatment in *C. roseus* .

**Dataset: S7.** The coexpression network of vindoline metabolism under ETH or MJ treatment in *C. roseus* .

**Dataset: S8.** The gene regulatory network of catharanthine and anhydrovinblastine metabolism by GENIE3.

**Dataset: S9.** The gene regulatory network of vindoline metabolism by GENIE3.

**Dataset: S10.** New‐old‐genome annotation.

## Data Availability

The raw RNA‐sequencing (RNA‐Seq) data has been submitted to the NCBI Sequence Read Archive under accession number GSE252019 (https://www.ncbi.nlm.nih.gov/geo/query/acc.cgi?acc=GSE252019). Gene sequences have been deposited in the GeneBank database under the accession numbers CrEIN3 (OR936141) and CrEIL1 (OR936142). All the data needed to evaluate the conclusions in the paper are present in the paper and/or the [Link], [Link], [Link], [Link], [Link], [Link], [Link], [Link], [Link], [Link], [Link].
