# Supplementary material for: Combined Transcriptomic and Metabolomic Analysis Reveals an Ethylene‐Activated Regulatory Model on Monoterpenoid Indole Alkaloid Biosynthesis in Catharanthus roseus
Source: Plant Biotechnol J. 2025 Sep 8;24(2):363–80. doi: 10.1111/pbi.70343 (PMC12906820; doi:10.1111/pbi.70343)
Supplement: Supplementary file 1 — Figure S1: NMR spectrum of C. roseus plants. Figure S2: Differential gene expression analysis. Figure S3: qPCR and HCL analysis of key genes in TIA biosynthetic pathway under MJ and ETH treatment. Figure S4: WGCNA analysis and co‐expression network. Figure S5: Complementation of Arabidopsis ein3eil1 mutantion by CrEIN3 and CrEIL1 genes. Figure S6: Silencing efficiency of CrEIN3 and CrEIL1 via VIGS. Figure S7: Phylogenetic analysis of CrERF5 and other known EIN3/EIL TFs from ten plant species. Figure S8: The calibration curves, linear regression equation and correlation coefficient (R2) of tryptamine, secologanin, ajmalicine, catharanthine, vindoline, anhydrovinblastine and vinblastine. Figure S9: Western blotting results of CrEIL1‐GFP overexpressed petals and CrEIN3‐GFP overexpressed petals. Figure S10: Regulatory effect of CrEIL1 on TIAs biosynthesis in C. roseus . Figure S11: Yeast one‐hybrid assays of CrEIN3 with the ORCA3, SAT, CS, TS and PRX1 promoters. Figure S12: Transient Dual‐luciferase assay of CrEIL1 with TIAs pathway genes promoters in tobacco. Figure S13: Co‐expression network for CrEIL1. Figure S14: Yeast two‐hybrid (Y2H) confirmed that CrEIL3 interacted with BIS2. Figure S15: Western blotting results of BIS2‐GFP overexpressed petals and BIS2‐GFP/CrEIL1 co‐overexpressed petals. Figure S16: Subcellular localization of BIS2‐GFP and BIS2‐GFP/CrEIL1 in tobacco leaves. Table S1: List of primers used in this work. Table S2: 1H NMR chemical shifts (δ) and coupling constants (Hz) of identified metabolites based on 1H‐NMR, J‐resolve, COSY, HSQC and references. Table S3: MIAs and precursors identified in Catharanthus roseus by UPLC‐Q/TOF MS. Table S4: Statistical analysis for selected signals from the NMR spectrum of ethylene treated, MeJA treated and control samples. [file PBI-24-363-s009.docx]

**SUPPLEMENTAL INFORMATION**

**Supplemental datasets**

Dataset S1 Untargeted and targeted metabolites content

Dataset S2 Summary of transcriptome mapping under ETH and MJ treatments in Catharanthus roseus

Dataset S3 Gene expression of C. roseus under ETH and MJ treatments

Dataset S4 Summary of co-expression gene modules.

Dataset S5 Trait modules correlation

Dataset S6 The coexpression network of catharanthine and anhydrovinblastine metabolism under ETH or MJ treatment in *C. roseus*.

Dataset S7 The coexpression network of vindoline metabolism under ETH or MJ treatment in *C. roseus*.

Dataset S8 The gene regulatory network of catharanthine and anhydrovinblastine metabolism by GENIE3.

Dataset S9 The gene regulatory network of vindoline metabolism by GENIE3.

Dataset S10 New-old-genome annotation

**Supplemental Figures**

Fig. S1 NMR spectrum of C. roseus plants.

Fig. S2 Differential gene expression analysis.

Fig. S3 qPCR and HCL analysis of key genes in TIA biosynthetic pathway under MJ and ETH treatment.

Fig. S4 WGCNA analysis and co-expression network.

Fig. S5 Complementation of Arabidopsis *ein3eil1* mutantion by *CrEIN3* and *CrEIL1* genes.

Fig S6 Silencing efficiency of *CrEIN3* and *CrEIL1* *via* VIGS.

Fig S7 Phylogenetic analysis of CrERF5 and other known EIN3/EIL TFs from ten plant species.

Fig. S8 The calibration curves, linear regression equation and correlation coefficient (R^2^) of tryptamine, secologanin, ajmalicine, catharanthine, vindoline, anhydrovinblastine and vinblastine.

Fig S9 Western blotting results of CrEIL1-GFP overexpressed petals and CrEIN3-GFP overexpressed petals.

Fig. S10 Regulatory effect of CrEIL1 on TIAs biosynthesis in *C. roseus*.

Fig S11 Yeast one-hybrid assays of CrEIN3 with the *ORCA3, SAT, CS, TS,* and *PRX1* promoters.

Fig. S12 Transient Dual-luciferase assay of CrEIL1 with TIAs pathway genes promoters in tobacco.

Fig. S13 Co-expression network for *CrEIL1*.

Fig. S14 Yeast two-hybrid (Y2H) confirmed that CrEIL3 interacted with BIS2.

Fig S15 Western blotting results of BIS2-GFP overexpressed petals and BIS2-GFP/CrEIL1 co-overexpressed petals.

Fig S16 Subcellular localization of BIS2-GFP and BIS2-GFP/CrEIL1 in tobacco leaves.

**Supplemental Tables**

Table S1. List of primers used in this work.

Table S2. ^1^H NMR chemical shifts (δ) and coupling constants (Hz) of identified metabolites based on ^1^H-NMR, *J*-resolve, COSY, HSQC and references

Table S3. MIAs and precursors identified in *Catharanthus roseus* by UPLC-Q/TOF MS.

Table S4 Statistical analysis for selected signals from the NMR spectrum of ethylene treated, MeJA treated and control samples.

Fig. S1


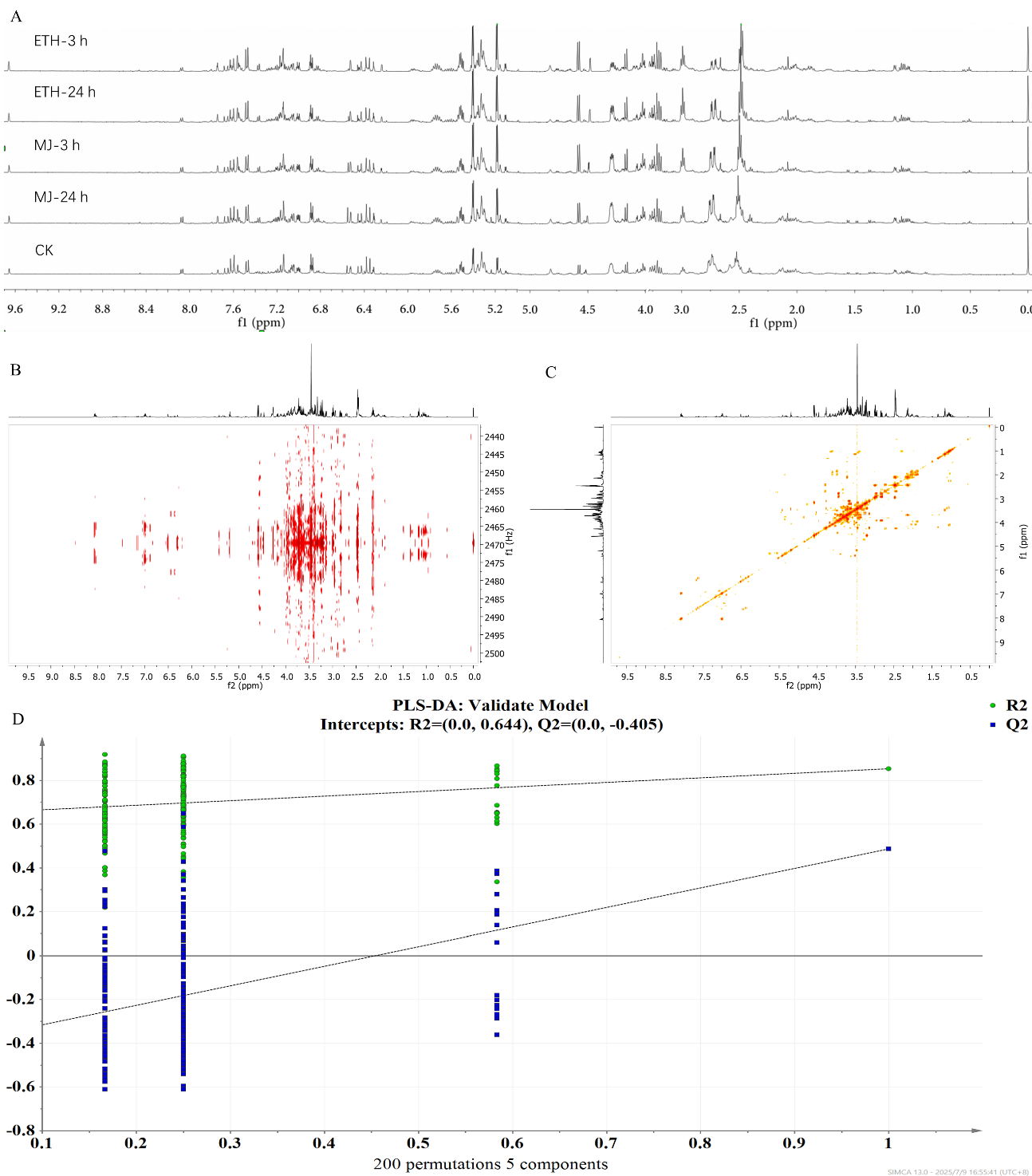


Fig. S1 NMR spectrum of C. roseus plants. (A) ^1^H-NMR spectrum of C. roseus plants under ETH or MJ treatments; (B) *J*-resolve, (C) COSY spectra of *C. roseus* and permutation test of PLS-DA (D): Green circle, R2 ; blue square, Q2. R2 = 0.644 and Q2 = -0.404.

Fig. S2


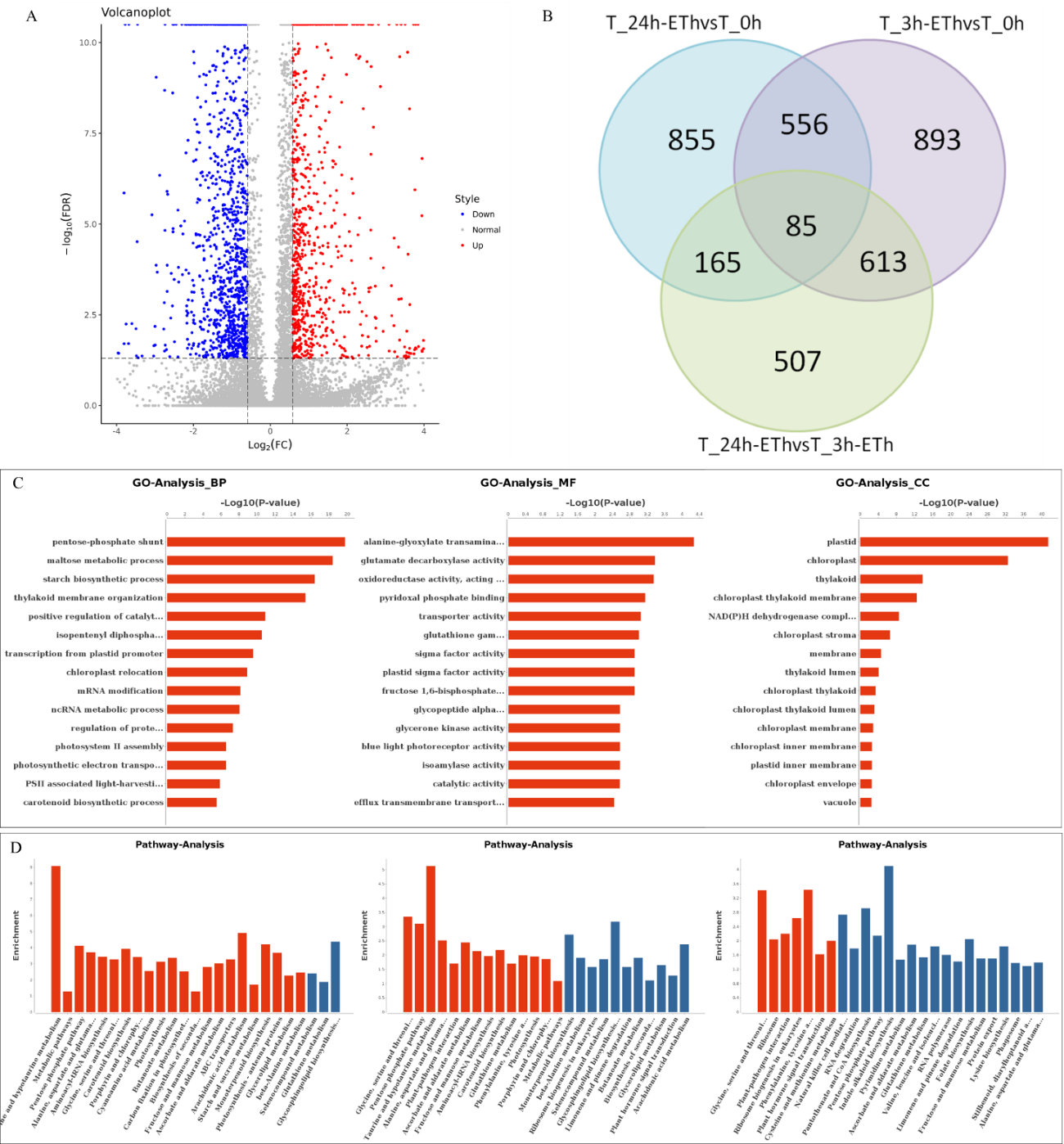


Fig. S2 Differential gene expression analysis. (A) Volcano plot of differential gene expression; (B) Venn analysis shows the number of the differentially expressed genes in three treatments; (C) Gene Ontology (GO) functional classification of assembled unigenes; (D) Pathway analysis of the differentially expressed genes according to KEGG database.

We applied EBSeq algorithm to filter the differentially expressed genes, after the significant analysis, P-value and FDR analysis were subjected to the following criteria: i) Fold Change>1.5 or < 0.667; ii), P-value<0.05, FDR<0.05. Gene ontology (GO) analysis was performed to facilitate elucidating the biological implications of the differentially expressed genes in the experiment. We downloaded the GO annotations from NCBI (http://www.ncbi.nlm.nih.gov/), UniProt (http://www.uniprot.org/) and the Gene Ontology (http://www.geneontology.org/). Fisher’s exact test was applied to identify the significant GO categories (P-value < 0.05). Pathway analysis was used to find out the significant pathway of the differentially expressed genes according to KEGG database. We turn to the Fisher’s exact test to select the significant pathway, and the threshold of significance was defined by P-value< 0.05.

Fig. S3


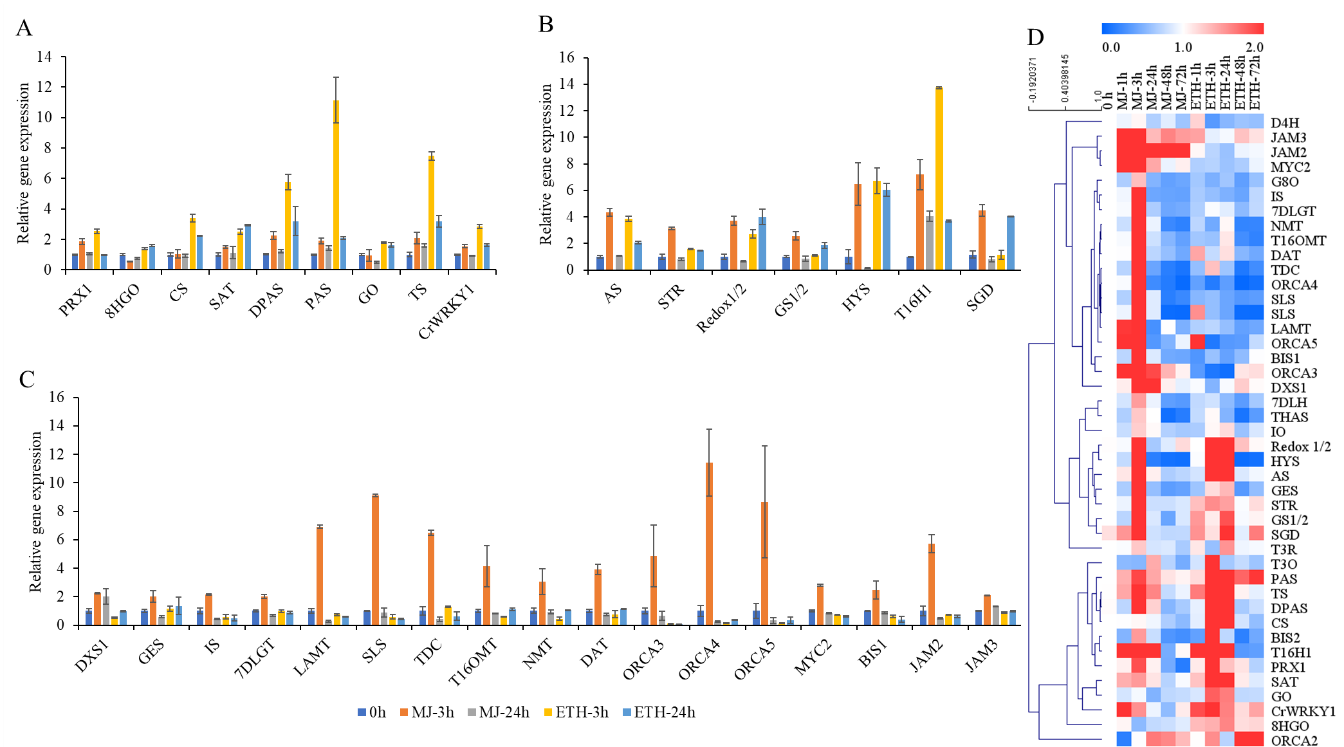


Fig. S3 qPCR and HCL analysis of key genes in MIA biosynthetic pathway under MJ and ETH treatment. (A) Group I genes that expression levels induced by ethylene signal; (B) Group II genes that expression levels induced by both ethylene and jasmonate signals; (C) Group III genes that expression levels mainly induced by jasmonate signal; (D) HCL analysis of MIA pathway genes and regulatory genes expression at 0 h, 1 h, 3 h, 24 h, 48 h and 72 h under MJ and ETH treatment *via* qPCR. The color scale (blue to red) represents the fold changes of each genes expression levels at different time points under ETH or JA treatment compared to the control at 0 h.

Fig. S4


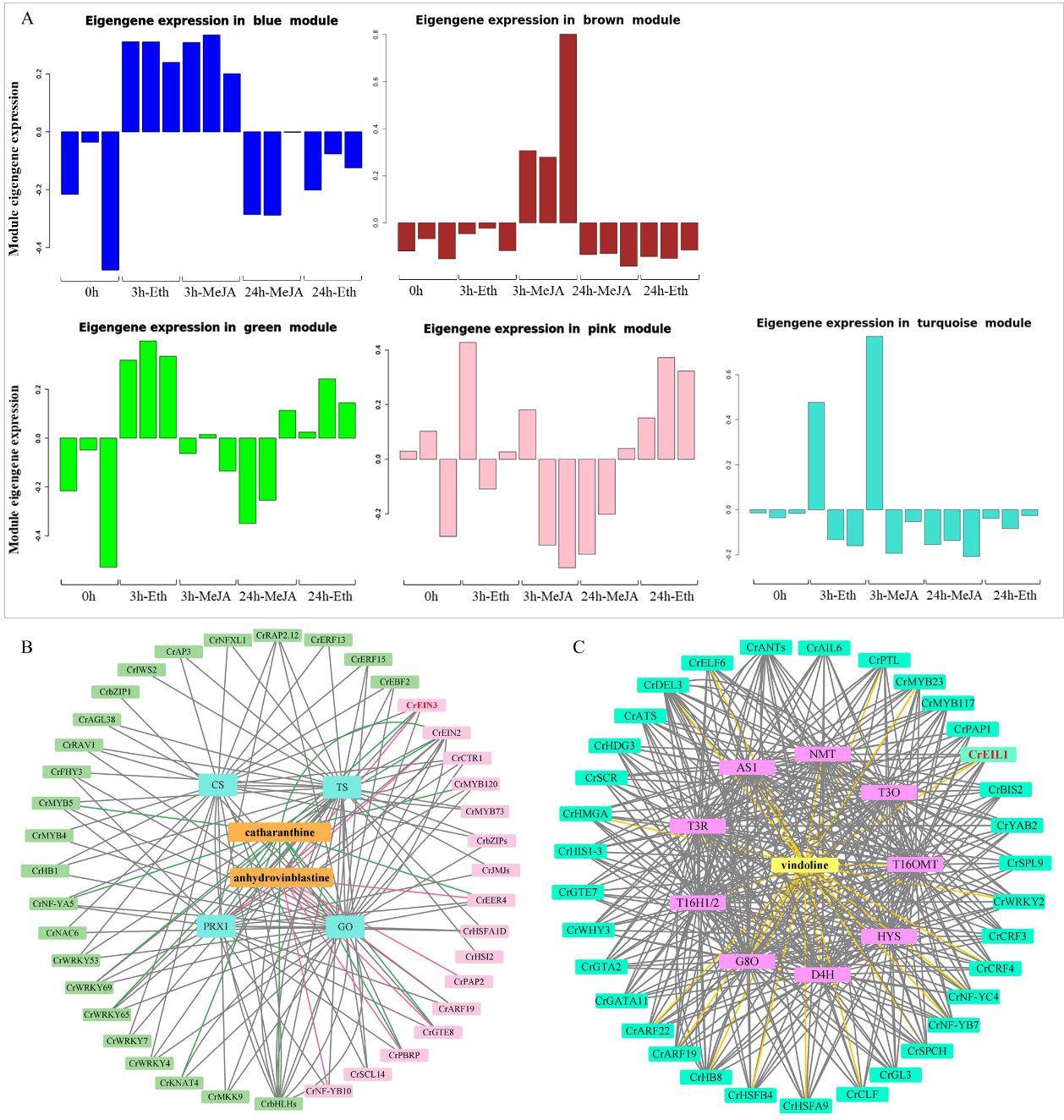


Fig. S4 WGCNA analysis and co-expression network. (A) Scaled MEs Expression Of blue, brown, green, pink and turquoise modules; (B) The co-expression network of anhydrovinblastine and catharanthine from the green and pink modules. Green boxes represent TFs from the green module. Pink boxes represent TFs from the pink module. Turquoise boxes represent MIA enzymic genes correlated with the TFs in the green and pink modules. (C) The co-expression network of vindoline from the turquoise module. Light turquoise boxes represent TFs from the turquoise module. Deep pink boxes represent MIA enzymic genes correlated with the TFs in the turquoise modules.

Weighted gene co-expression network analysis (WGCNA) was performed across samples using the standard method with a power of 16 to cluster the gene expression patterns. The TFs was defined by tblastn with arabidopsis and rice. We then constructed the co-expression networks (weight > 0.05) based on the TFs and their related genes. The transcriptional regulatory networks were generated by combining the Pearson correlation coefficient (PCC > 0.8) between structural genes and transcription factors in the same module. The networks were visualized by CYTOSCAPE (v.3.9.1, USA).

Fig. S5


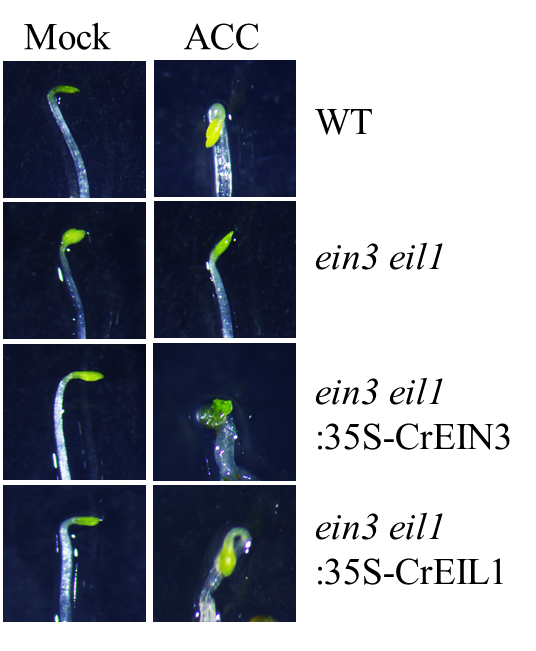


Fig. S5 Complementation of Arabidopsis *ein3eil1* mutantion by *CrEIN3* and *CrEIL1* genes.

The hook phenotypes of 4-d-old etiolated Arabidopsis seedlings Col-0 (WT), *ein3 eil1*, *ein3 eil1*:35S-CrEIN3, and *ein3 eil1*:35S-CrEIL1 grown in the dark on MS medium supplied without (Mock) or with 10 mM ACC.

Figure S6


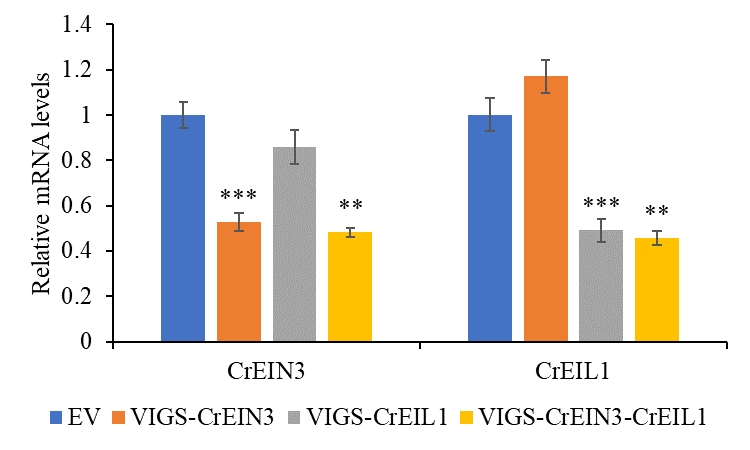


Fig S6 Silencing efficiency of *CrEIN3* and *CrEIL1* *via* VIGS. The error bars represent the means ± SD (standard deviation) from three replicates. Asterisks indicate statistically significant differences using Student’s t-test. *P < 0.05, **P < 0.01, ***P < 0.001.

Figure S7


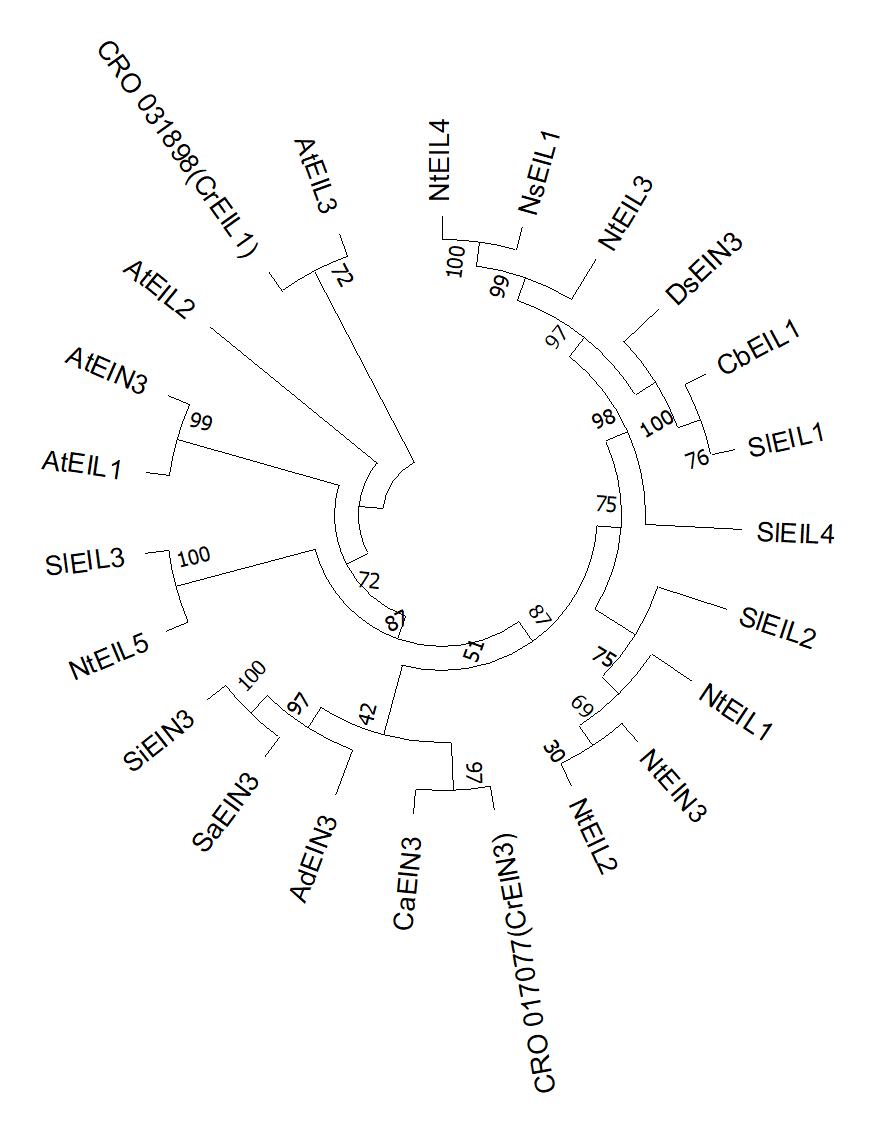


Fig S7 Phylogenetic analysis of CrERF5 and other known EIN3/EIL TFs from ten plant species. *Arabidopsis* (At)*, Solanum lycopersicum* (Sl)*, Nicotiana tabacum* (Nt)*, Coffea arabica* (Ca)*, Sesamum indicum* (Si)*, Sesamum alatum* (Sa)*, Abeliophyllum distichum* (Ad)*, Capsicum baccatum* (Cb)*, Datura stramonium* (Ds)*, Nicotiana sylvestris* (Ns). The tree presented here is a neighbor-joining tree based on amino acid sequence alignment.

Accession numbers

Gene sequences have been deposited in the GeneBank database under the accession numbers CaEIL1 ([XP_027081706.1](https://www.ncbi.nlm.nih.gov/protein/XP_027081706.1?report=genbank&log$=prottop&blast_rank=6&RID=5MGKS98Y016)), CaEIN3 (AHA93899.1), SiEIN3 (XP_011080514.1), DsEIN3 (MCD7455382.1), SaEIN3 (KAK4419709.1), DsEIN3 (MCE5165812.1), AdEIN3 (KAL2485533.1), NsEIL1 (XP_009798829.1), CbEIL1 (PHT46472.1)

Fig S8


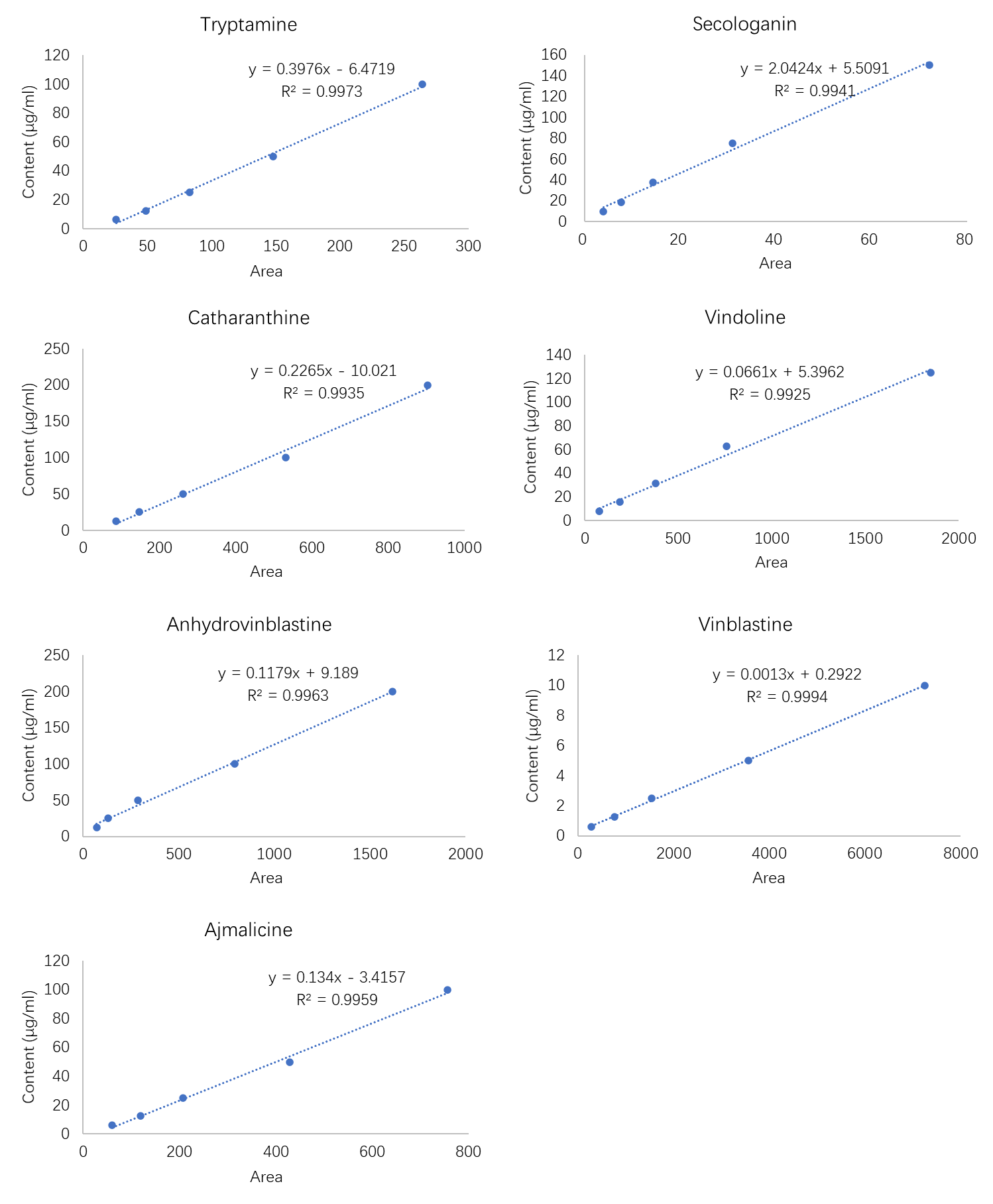


Figure S8 The calibration curves, linear regression equation and correlation coefficient (R^2^) of tryptamine, secologanin, ajmalicine, catharanthine, vindoline, anhydrovinblastine and vinblastine.

UPLC-Q/TOF MS analyses were performed using a Primer UPLC-Q-TOF mass spectrometer (Waters Corp., Milford, MA, United States) equipped with an electrospray ionization source. Data acquisition, handling and instrument control were performed using MassLynx 4.1 software. Mass range, m/z 50–1000 in positive mode; capillary, 3.0 kV; sample cone voltage, 35V; extraction cone, 3.0; ion guide, 3.0; source temperature, 115◦C; desolvation gas temperature, 300◦C; flow rate of desolvation gas, 700 L h−1.

To ensure accuracy and reproducibility, all analyses were conducted using an independent reference spray via the Lock Spray interface; Tyr–Gly–Gly–Phe–Leu (leucine-encephalin, 200 pg mL−1) was used as a lock mass (m/z 556.2771) under positive-ion conditions for real-time calibration (flow rate of 30 mL min−1). Before the experiment, a single-point calibration was performed against the lock mass compound (leucineencephalin). A multiple-point calibration was then performed over the range m/z 50–1000 using sodium formate solution (prepared from 10% formic acid/0.1 mol L−1 sodium hydroxide solution/acetonitrile, 10 mL/10 mL/80 mL). All points fell within 1 ppm during calibration. The resolving power of the instrument was 8000.

UPLC conditions: a BEH C18 (100 mm × 2.1 mm, 1.7 mm) column (Waters) was used; the column temperature was maintained at 40◦C. Mobile phases A (water, 0.1% formic acid) and B (acetonitrile, 0.1% formic acid) were used; the gradient program was as follows: 0−4 min 5–25% B, 9–12 min 45–85% B, 14 min 100% B; 14.5–16 min 5% B, flow rate 0.35 mL min−1; injection volume 2 mL. Acquity PDA detector wavelength was fixed at 210, 254, and 278 nm. A mixture of reference standards of MIAs and precursors (tryptamine, secologanin, ajmalicine, catharanthine, and vinblastine were purchased from Sigma-Aldrich, St. Louis, MO, United States; vindoline and anhydrovinblastine were purchased from Shanghai R&D Center for Standardization of Chinese Medicines, China) were detected and identified based on MS/MS spectra (Table S3). Seven standard stock solutions with a concentration of 1mg/mL were prepared in methanol. A series of calibration working solutions were obtained by diluting the stock solution with methanol. The concentrations of calibration working solutions of tryptamine and ajmalicine standards were set up to 100 μg/mL, 50 μg/mL, 25 μg/mL, 12.5 μg/mL, 6.25 μg/mL. The concentrations of calibration working solutions of catharanthine and anhydrovinblastine standards were set up to 200 μg/mL, 100 μg/mL, 50 μg/mL, 25 μg/mL, 12.5 μg/mL. The concentrations of calibration working solutions of secologanin standards were set up to 150 μg/mL, 75 μg/mL, 37.5 μg/mL, 18.25 μg/mL, 9.375 μg/mL. The concentrations of calibration working solutions of vindoline standards were set up to 125 μg/mL, 62.5 μg/mL, 31.25 μg/mL, 15.625 μg/mL, 7.8125 μg/mL. The concentrations of calibration working solutions of vinblastine standards were set up to 10 μg/mL, 5 μg/mL, 2.5 μg/mL, 1.25 μg/mL, 0.625 μg/mL. Samples were applied in triplicate for quantification using calibration curves of the standards.

Figure S9


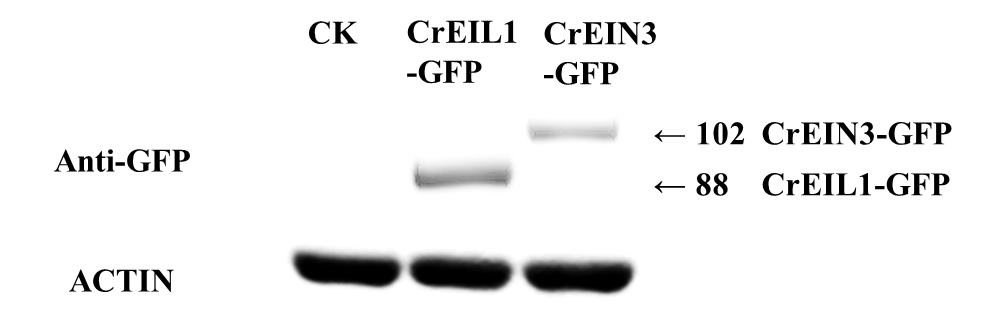


Fig S9 Western blotting results of CrEIL1-GFP overexpressed petals and CrEIN3-GFP overexpressed petals. CK was the control transformed with pHB-GFP vector.

Fig S10


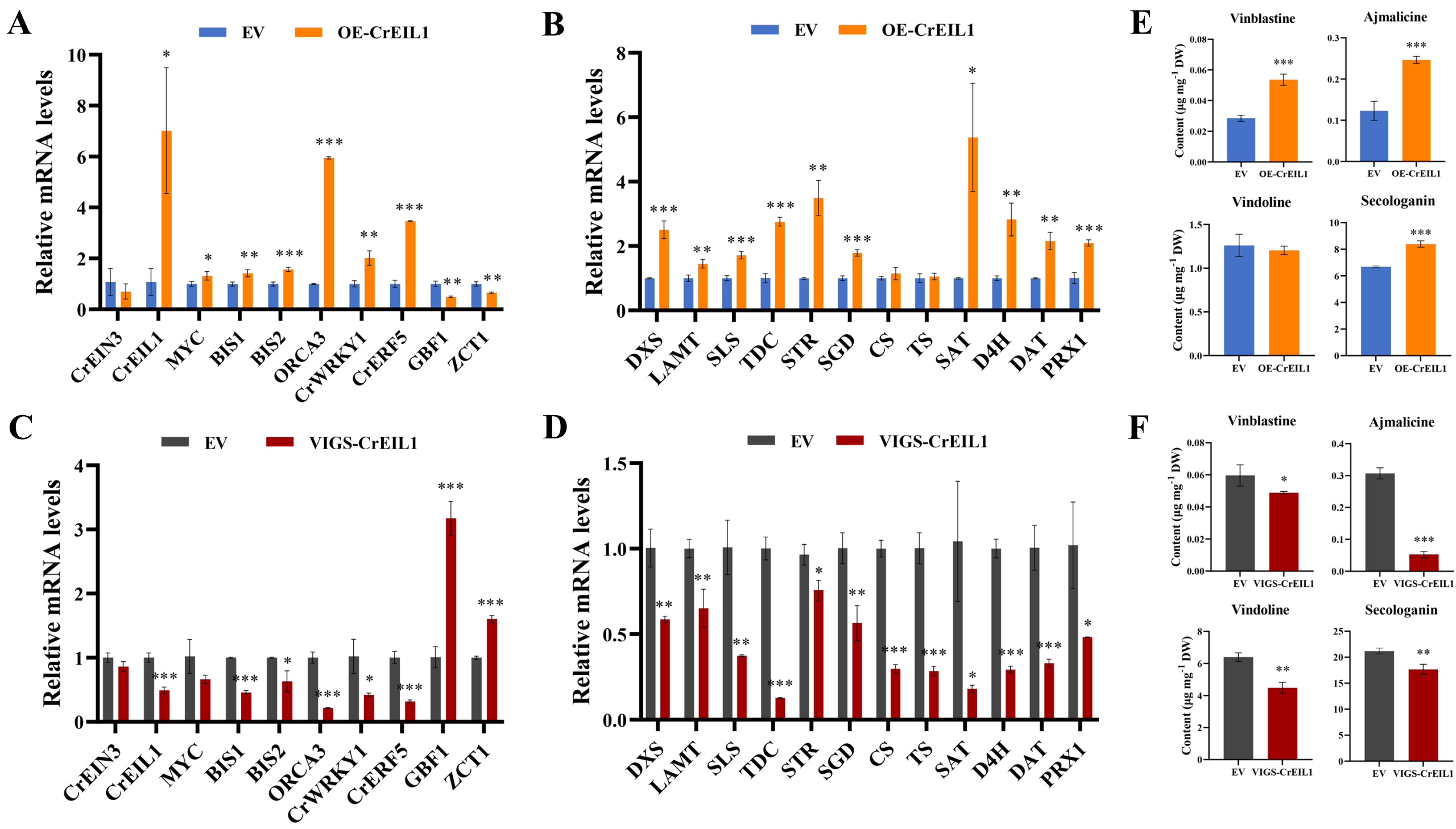


Figure S9 Regulatory effect of CrEIL1 on TIAs biosynthesis in *C. roseus*.

(A, B) Gene expression levels of TIAs related genes in *CrEIL1* overexpression lines. (C, D) Gene expression levels of TIAs related genes in *CrEIL1* silencing lines. (E) TIAs contents in *CrEIL1* overexpression lines. (D) TIAs contents in *CrEIL1* silencing lines. EV, empty vector; OE, overexpression; VIGS, virus induced gene silence. The error bars represent the means ± SD (standard deviation) from three replicates. Asterisks indicate statistically significant differences using Student’s t-test. ^*^ *P* < 0.05.

Fig. S11


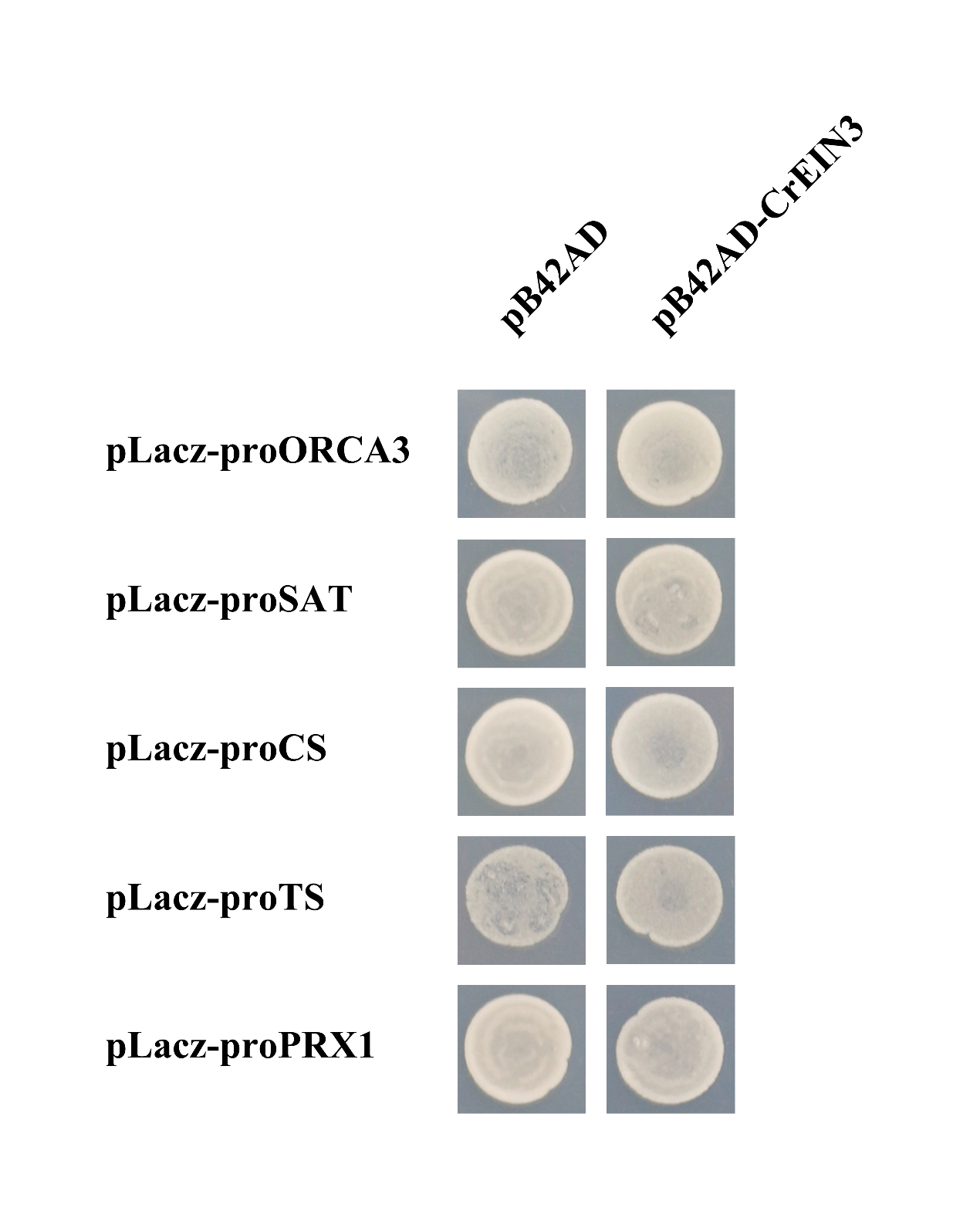


Fig S11 Yeast one-hybrid assays of CrEIN3 with the *ORCA3, SAT, CS, TS,* and *PRX1* promoters. EGY48A yeast expressing pB42AD or pB42AD-CrEIN3 and ATGTA-box fragments from gene promoters were grown on SD/−Ura/−Trp medium (20 mg l−1 X-gal). The white plaques showed negative protein–DNA interactions.

Fig S12


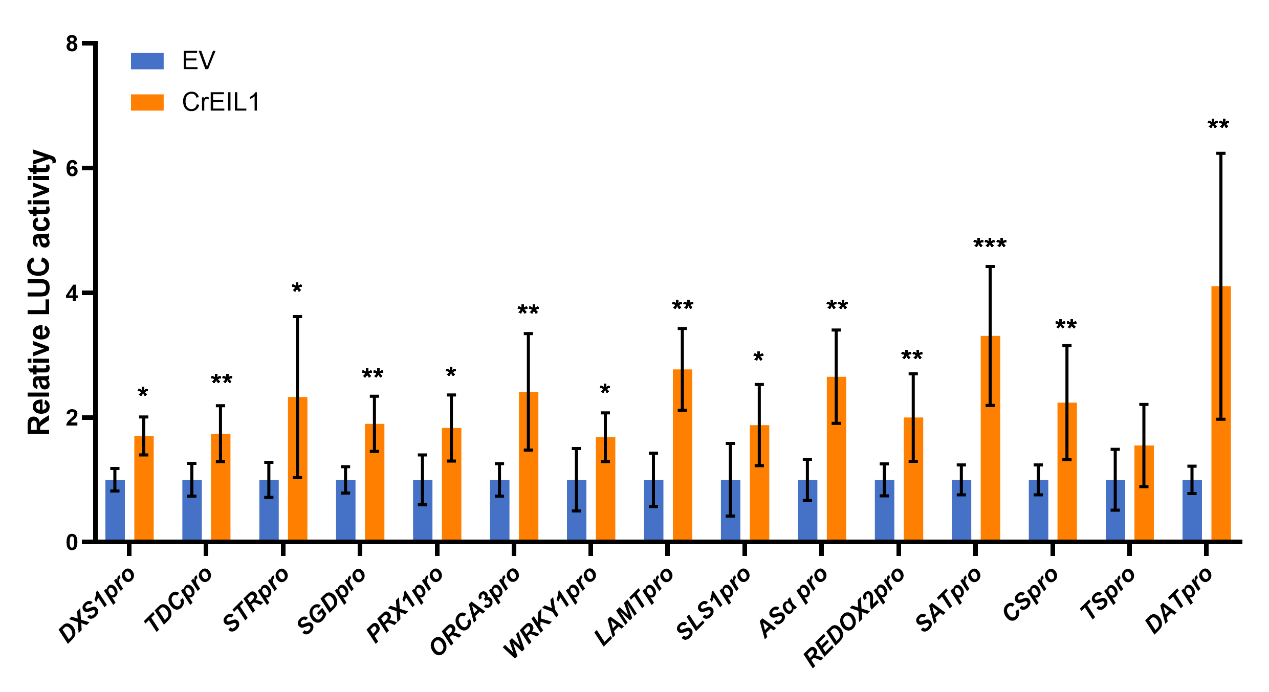


Fig. S12. Transient Dual-luciferase assay of CrEIL1 with MIAs pathway genes promoters in tobacco. The LUC/REN values for the combination of empty effector construct p2300 and each reporter construct were set to 1. The error bars represent the means ± SD from three biological replicates, and asterisks indicate statistically significant differences compared with the controls. *P < 0.05, **P < 0.01, ***P < 0.001.

Fig. S13


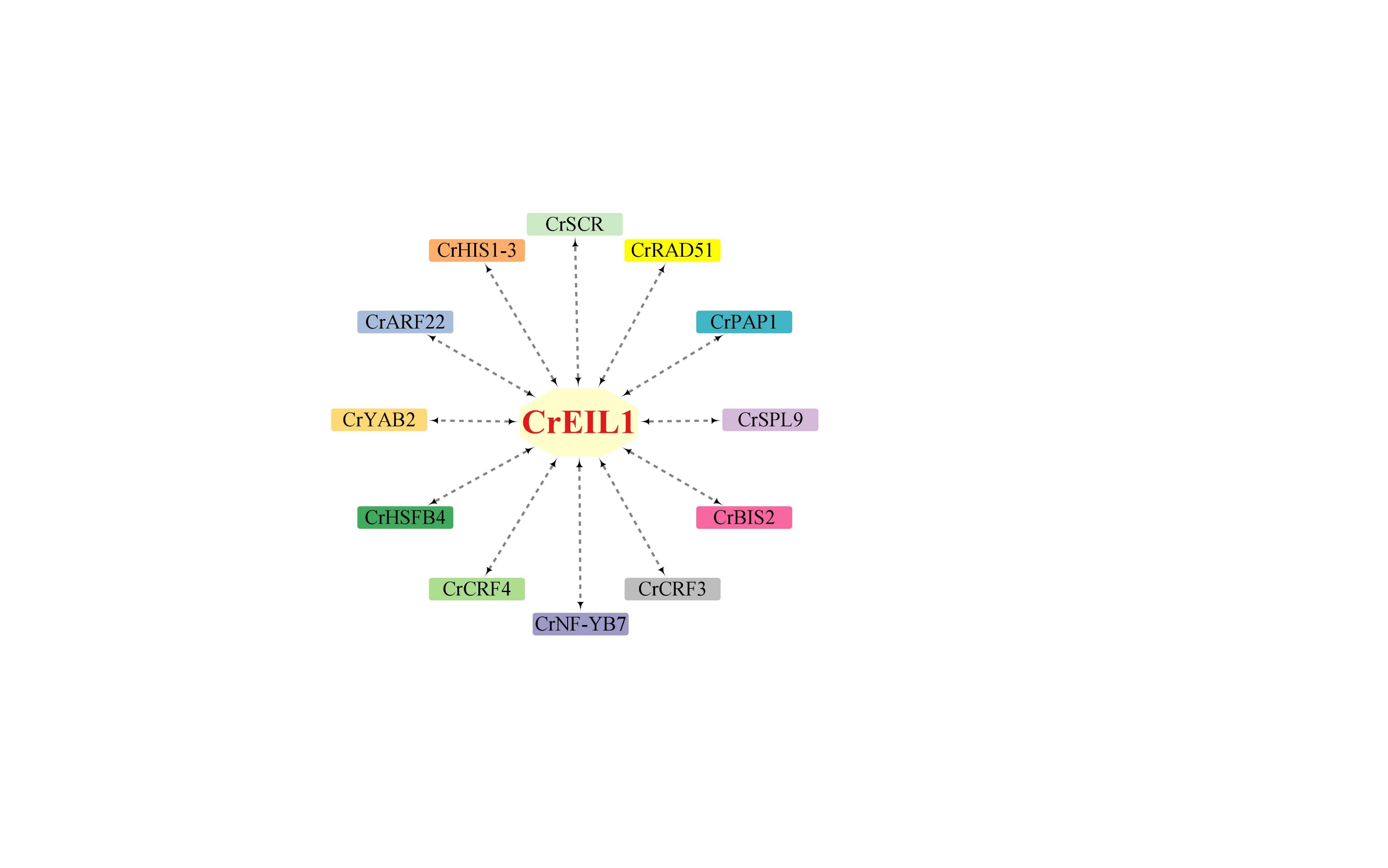


Fig. S13 Co-expression network for *CrEIL1*. Circles with different colors represents different families of transcription factors identified as highly correlated with *CrEIL1* expression.

Fig. S14


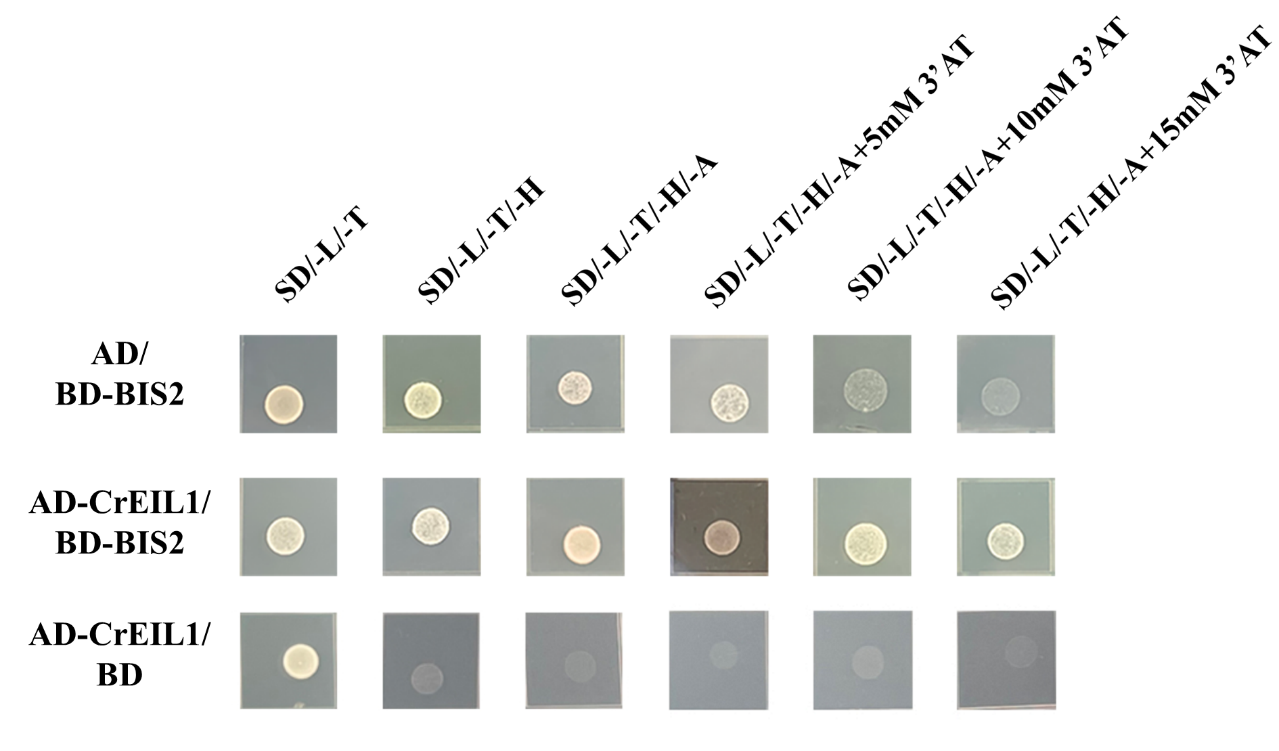


Fig. S14 Yeast two-hybrid (Y2H) confirmed that CrEIL3 interacted with BIS2. Transformed yeast cells were grown on control medium SD/-Trp/-Leu, and selective media SD/-Trp/-Leu/-His and SD/-Trp/-Leu/-His/-Ade+15mM 3’AT. Pictures were taken after 3 days of incubation at 30℃. Y2H assays were repeated three times. pGADT7 plasmid was used for negative control. Representative results are shown.

Fig S15


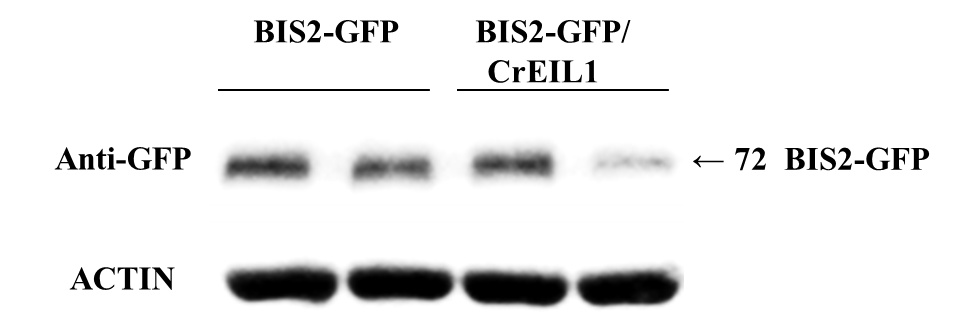


Fig S15 Western blotting results of BIS2-GFP overexpressed petals and BIS2-GFP/CrEIL1 co-overexpressed petals.

Fig S16


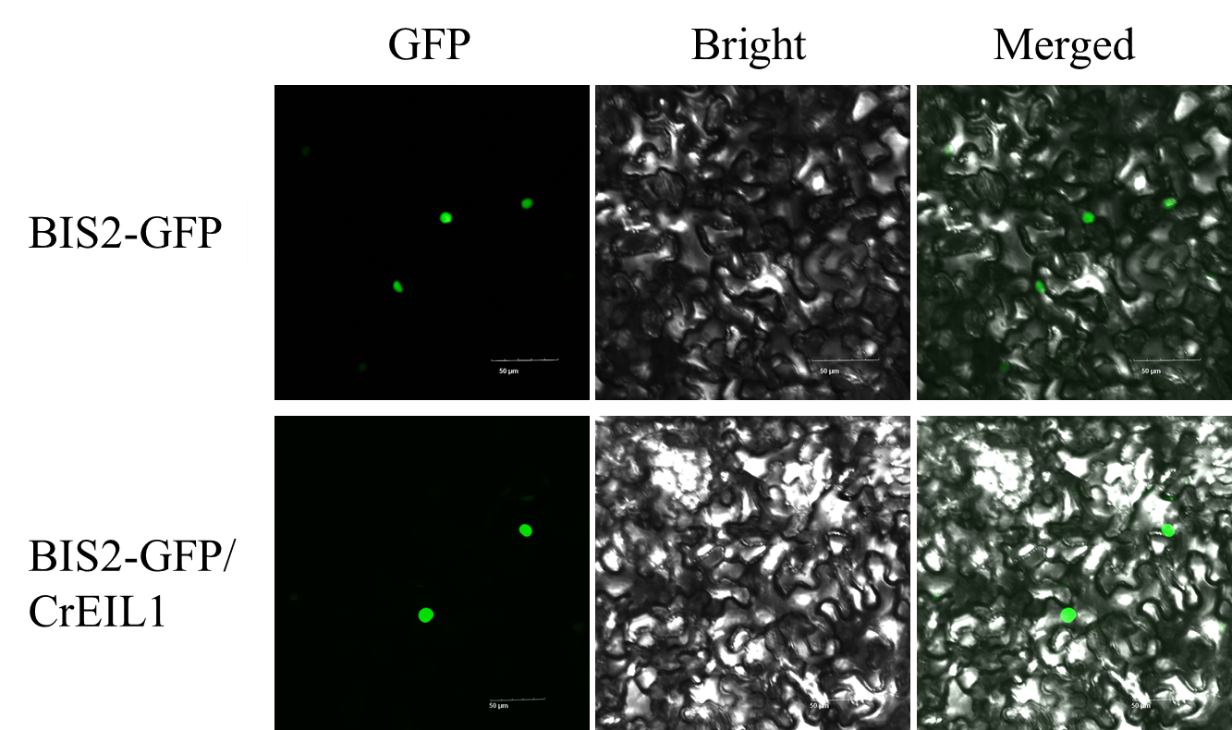


Fig S16 Subcellular localization of BIS2-GFP and BIS2-GFP/CrEIL1 in tobacco leaves. Bars = 50 μm.

Table S1. List of primers used in this work.

| Primers | Purpose | Primer Sequence (5’-3’) |
| --- | --- | --- |
| pLB-CrEIL1-FP | Cloning | GAGGGTCTCTGAATTTGTTG |
| pLB-CrEIL1-RP | Cloning | CCTCCTTGTTCATGAACTGC |
| pLB-CrEIL3-FP | Cloning | GATATCCTTTGCGTAAATCC |
| pLB-CrEIL3-RP | Cloning | CCTCCTTGTTCATGAACTGC |
| pLB-BIS2-FP | Cloning | GCAGGCTGATGATTTATTCCCT |
| pLB-BIS2-RP | Cloning | CCCCATCATCTTCTTTCTTCCT |
| pHB-CrEIL1-GFP-FP | Construction | CTCAAGCTTGGATCCATGATGATGTTTGAGGAAAT |
| pHB-CrEIL1-GFP-RP | Construction | GCTCACCATACTAGTCGGATACCAGATTGGAATAT |
| pHB-CrEIL3-GFP-FP | Construction | CTCAAGCTTGGATCCATGGTGAAGTTTGATGAAGA |
| pHB-CrEIL3-GFP-RP | Construction | GCTCACCATACTAGTTGGATCTCCTTGGTGAAAAT |
| N2227-F | qRT-PCR | GGTTGCTCTTCATTACGGATTT |
| N2227-R | qRT-PCR | TGCAGCATAGTAATGGTTTTGC |
| CrEIL1-RT-FP | qRT-PCR | CTGTCCACCTTTGTCCTCAT |
| CrEIL1-RT-RP | qRT-PCR | ATGTGGTTGTGGTTGACCTG |
| CrEIL3-RT-FP | qRT-PCR | ACAACTACCGGATTGCATGG |
| CrEIL3-RT-RP | qRT-PCR | TCAAGTTCGTCCTGTTTCCAC |
| CrWRKY1-F | qRT-PCR | GTACTTGGTCCCGACGATATTC |
| CrWRKY1-R | qRT-PCR | CGAAACATTCCTTCGTTTGTAAG |
| CrMYC2-F | qRT-PCR | AGTGGTGAAGGAGGCAGAGA |
| CrMYC2-R | qRT-PCR | ATGGCTCTTCCCTTCCATTT |
| ORCA3-F | qRT-PCR | CGGGATCCGAAATACAGAAA |
| ORCA3-R | qRT-PCR | GCCCTTATACCGGTTCCAAT |
| CrBIS1-F | qRT-PCR | ATGGAATCAGTGGTGCTAGTGA |
| CrBIS1-R | qRT-PCR | TTCAATTTCAGGGAGCTGTGAC |
| CrBIS2-F | qRT-PCR | TGGCTCTTTCTACTCTAGTTCCC |
| CrBIS2-R | qRT-PCR | CACCACCGATTCCATAGTTTG |
| CrZCT1-F | qRT-PCR | AGCCGAAAACTCATGCTTGT |
| CrZCT1-R | qRT-PCR | CGCCTTTGCAACAGGTTTAT |
| CrGBF1-F | qRT-PCR | AACAGGCTGAGACGGAAGAA |
| CrGBF1-R | qRT-PCR | GACCCGTGCATTTTTCAACT |
| DXS1-F | qRT-PCR | TCGCTGCAGAACTTAGAGCA |
| DXS1-R | qRT-PCR | GCCAACATCCCAAATGATTC |
| TDC-F | qRT-PCR | ATCCGATCAAACCCATACCA |
| TDC-R | qRT-PCR | CGTCATCCTCGACCATTTTT |
| STR-F | qRT-PCR | ACCATTGTGTGGGAGGACAT |
| STR-R | qRT-PCR | ATTTGAATGGCACTCCTTGC |
| SGD-F | qRT-PCR | GGAGGCTTCTTGAGTGATCG |
| SGD-R | qRT-PCR | GCAAATTCACCAGTGGCATA |
| PRX1-F | qRT-PCR | ATTCAAGGTGCCCTGACTTG |
| PRX1-R | qRT-PCR | TGACGGAGAGTCAAGTTAGG |
| SLS-F | qRT-PCR | GTTCCTTCTCACCGGAGTTG |
| SLS-R | qRT-PCR | CCCATTTGGTCAACATGTCA |
| DAT-F | qRT-PCR | CTTCTTCTCATCACGTACCAACTC |
| DAT-R | qRT-PCR | ATACCAAACTCAACGGCCTTAG |
| SAT-F | qRT-PCR | GGATGGGGAAAGCCTGTTTCTGTT |
| SAT-R | qRT-PCR | CTTCAGCCATGCTGATCCATGCTT |
| HL1-F | qRT-PCR | TGGGGCTGGCTTTTGTCTAGAATC |
| HL1-R | qRT-PCR | TAAGCTGCGGGTAAAAGGTGCTCT |
| HL2-F | qRT-PCR | TGCTCCTGGTGGAAATGATAACCC |
| HL2-R | qRT-PCR | AATCAGCAACCTCGAGCAACCA |
| pGreen-SGD-FP | Cloning | CGGTATCGATAAGCTTAAACCGTGGAGGTTCATTAT |
| pGreen-SGD-RP | Cloning | ATCCCCCGGGCTGCAGGAACTATAATGAATATATAT |
| pGreen-STR-FP | Cloning | CGGTATCGATAAGCTTCTGAAAGTGAAAGTTTTAGT |
| pGreen-STR-RP | Cloning | ATCCCCCGGGCTGCAGGTACCACGTGGAGTAATATT |
| pGreen-TDC-FP | Cloning | CGGTATCGATAAGCTTGGATCTCAAGTTTAAGGAAA |
| pGreen-TDC-RP | Cloning | ATCCCCCGGGCTGCAGCTGGTGTAGGTGTATGTGTA |
| pGreen-DXS1-FP | Cloning | CGGTATCGATAAGCTTCGTATTATGCCCCTACATCC |
| pGreen-DXS1-RP | Cloning | ATCCCCCGGGCTGCAGATGGAGATTTTGGATTGGAG |
| pGreen-PRX1-FP | Cloning | CGGTATCGATAAGCTTTCGACCAAGGAAAGCTTGAG |
| pGreen-PRX1-RP | Cloning | ATCCCCCGGGCTGCAGAGGAAGGGAGGAAAAAAC |
| pGreen-7-DLGT-FP | Cloning | CGGTATCGATAAGCTTAATATGGACTTGGAACTGGG |
| pGreen-7-DLGT-RP | Cloning | ATCCCCCGGGCTGCAGATATCCATGACTCTTTCGCC |
| pGreen-7-DLH-FP | Cloning | CGGTATCGATAAGCTTCAAAGTAACAGAGAAGAAC |
| pGreen-7-DLH-RP | Cloning | ATCCCCCGGGCTGCAGTTCAGTCCAAAGTCAGGCA |
| pGreen-SLS-FP | Cloning | CGGTATCGATAAGCTTAAGACAACCTGACCTCCTCA |
| pGreen-SLS-RP | Cloning | ATCCCCCGGGCTGCAGATGAGGCGAGGAACAATATC |
| pGreen-LAMT-FP | Cloning | CGGTATCGATAAGCTTTAACGTCTCTTAAGCTGGTG |
| pGreen-LAMT-RP | Cloning | ATCCCCCGGGCTGCAGGAGTCATCACCACCTTTCAT |
| pGreen-ASa-FP | Cloning | CGGTATCGATAAGCTTCGAGCTTCAACTACTGGAAT |
| pGreen-ASa-RP | Cloning | ATCCCCCGGGCTGCAGCTTGATAAGACGAGGAGGTG |
| pGreen-SAT-FP | Cloning | CGGTATCGATAAGCTTGGATATCAACCGCCTACTTT |
| pGreen-SAT-RP | Cloning | ATCCCCCGGGCTGCAGTCGGAGATGATGGTTGAATC |
| pGreen-DAT-FP | Cloning | CGGTATCGATAAGCTTGCGTAGATGTATGATGATGC |
| pGreen-DAT-RP | Cloning | ATCCCCCGGGCTGCAGTACGCACGTTTGGTATATGT |
| pGreen-ORCA3-FP | Cloning | CGGTATCGATAAGCTTTACGCGTCACAAATTTCAAG |
| pGreen-ORCA3-RP | Cloning | ATCCCCCGGGCTGCAGAAGAAATCGATCTGAGACGG |
| pGreen-WRKY1-FP | Cloning | CGGTATCGATAAGCTTTCCCAATTCCCATAATTCAG |
| pGreen-WRKY1-RP | Cloning | ATCCCCCGGGCTGCAGAGATTTGTACGGAGAGTTCG |
| pGreen-HL1-FP | Cloning | CGGTATCGATAAGCTTGGTGTAGGCCAGTTTATTGA |
| pGreen-HL1-RP | Cloning | ATCCCCCGGGCTGCAGCTAGCGGAAACTTGTGATGA |
| pGreen-HL2-FP | Cloning | CGGTATCGATAAGCTTGATGAGTTAGGCCTAAACCC |
| pGreen-HL2-RP | Cloning | ATCCCCCGGGCTGCAGGTCTTTCCAAGAGACTCCAC |
| pGreen-REDOX2-FP | Cloning | CGGTATCGATAAGCTTGCTACTCTTATGTGGCTTCC |
| pGreen- REDOX2-RP | Cloning | ATCCCCCGGGCTGCAGGGTTAGTTCTTCCAACGGTG |
| pB42AD-CrEIL1-FP | Y1H | GCCTCTCCCGAATCCATGATGATGTTTGAGGAAAT |
| pB42AD-CrEIL1-RP | Y1H | CCAAAGCTTCTCGAGTTACGGATACCAGATTGGAA |
| pLacZ-SGD-241-FP | Y1H | AATTCGATACAATGTATGAATAGATACAATGTATGAATAGATACAATGTATGAATAC |
| pLacZ-SGD-241-RP | Y1H | TCGAGTATTCATACATTGTATCTATTCATACATTGTATCTATTCATACATTGTATCG |
| pLacZ-DXS1-694-FP | Y1H | AATTCCTTCAAATGTATTTGGACTTCAAATGTATTTGGACTTCAAATGTATTTGGAC |
| pLacZ-DXS1-694-RP | Y1H | TCGAGTCCAAATACATTTGAAGTCCAAATACATTTGAAGTCCAAATACATTTGAAGG |
| pLacZ-7DLH1-460-FP | Y1H | AATTCTAATTCATGTAACCAGTTAATTCATGTAACCAGTTAATTCATGTAACCAGTC |
| pLacZ-7DLH1-460-RP | Y1H | TCGAGACTGGTTACATGAATTAACTGGTTACATGAATTAACTGGTTACATGAATTAG |
| pLacZ-WRKY1-1367-FP | Y1H | AATTCAAATAAATGTATCCAACAAATAAATGTATCCAACAAATAAATGTATCCAACC |
| pLacZ-WRKY1-1367-RP | Y1H | TCGAGGTTAATTACATTAAAATGTTAATTACATTAAAATGTTAATTACATTAAAATG |
| pCold-CrEIL1-FP | EMSA | TACCCTCGAGGGATCCATGATGATGTTTGAGGAAAT |
| pCold-CrEIL1-RP | EMSA | AGATTACCTATCTAGATTACGGATACCAGATTGGAA |
| proSGD-probe-lable-FP | EMSA | AAATCCATAAGATACAATGTATGAATAACTATACCT |
| proSGD-probe-RP | EMSA | AGGTATAGTTATTCATACATTGTATCTTATGGATTT |
| proSGD-probe-FP | EMSA | AAATCCATAAGATACAATGTATGAATAACTATACCT |
| proSGD-probe-mutant-RP | EMSA | AGGTATAGTTATTCAAAAAATGTATCTTATGGATTT |
| proSGD-probe-mutant-FP | EMSA | AAATCCATAAGATACATTTTTTGAATAACTATACCT |
| AD-CrEIL3-FP | Y2H | GAGGCCAGTGAATTCATGGTGAAGTTTGATGAAG |
| AD-CrEIL3-RP | Y2H | GAGCTCGATGGATCCTTATGGATCTCCTTGGTGA |
| BD-CrWRKY1-FP | Y2H | ATGGAGGCCGAATTCATGAACACCACGATGGCTTG |
| BD-CrWRKY1-RP | Y2H | CAGGTCGACGGATCCTTACAAAAGAGTAGAATGTG |
| BD-ORCA3-FP | Y2H | ATGGAGGCCGAATTCATGTCCGAAGAAATCATTTC |
| BD-ORCA3-RP | Y2H | CAGGTCGACGGATCCTTAATATCGTCTCTTCTTCC |
| BD-CrMYC2-FP | Y2H | ATGGAGGCCGAATTCATGACGGACTATAGGCTACA |
| BD-CrMYC2-RP | Y2H | CAGGTCGACGGATCCTCATACCAAGAGCCTCATCG |
| BD-CrBIS1-FP | Y2H | ATGGAGGCCGAATTCATGACAATGATGATGACGAT |
| BD-CrBIS1-RP | Y2H | CAGGTCGACGGATCCTCAATCTTCTAAATTTGCAC |
| BD-CrBIS2-FP | Y2H | ATGGAGGCCGAATTCATGATGACGATGATGATGGA |
| BD-CrBIS2-RP | Y2H | CAGGTCGACGGATCCTCAGTCTGCCATTGGTGGAC |
| BD-CrJAZ1-FP | Y2H | ATGGAGGCCGAATTCATGGCTTCATCGGAGATGAT |
| BD-CrJAZ1-RP | Y2H | CAGGTCGACGGATCCTTAAAAAGGAAAGCCAATTTC |
| BD-CrZCT1-FP | Y2H | ATGGAGGCCGAATTCATGGGCGTGAAGAGATTCAG |
| BD-CrZCT1-RP | Y2H | CAGGTCGACGGATCCTTAGACGAAAAATTCCAGTA |
| BD-CrZCT2-FP | Y2H | ATGGAGGCCGAATTCATGGTGATGATTAATATACC |
| BD-CrZCT2-RP | Y2H | CAGGTCGACGGATCCTCATAAGAAGCAATCAACAG |
| BD-CrZCT3-FP | Y2H | ATGGAGGCCGAATTCATGGCACTTGAAGCTTTGAA |
| BD-CrZCT3-RP | Y2H | CAGGTCGACGGATCCCTAATTAATTTGATGGTTTTC |
| cYFP-CrEIL3-FP | BiFC | ATTACAGGTACCCGGGATGGTGAAGTTTGATGAAGA |
| cYFP-CrEIL3-RP | BiFC | CGCCGTCGACTCTAGATGGATCTCCTTGGTGAAAAT |
| nYFP-BCrEIL3-FP | BiFC | GGACGCCGGCGGATCCATGGTGAAGTTTGATGAAGA |
| nYFP-CrEIL3-RP | BiFC | AGCTCTGCAGGTCGACTGGATCTCCTTGGTGAAAAT |
| cYFP-CrBIS2-FP | BiFC | ATTACAGGTACCCGGGATGATGACGATGATGATGGA |
| cYFP-CrBIS2-RP | BiFC | CGCCGTCGACTCTAGAGTCTGCCATTGGTGGACTGT |
| nYFP-CrBIS2-FP | BiFC | GGACGCCGGCGGATCCATGATGACGATGATGATGGA |
| nYFP-CrBIS2-RP | BiFC | AGCTCTGCAGGTCGACGTCTGCCATTGGTGGACTGT |
| SGD promoter-F | ChIP | tcagactcttgacgacattcaga |
| SGD promoter-R | ChIP | tgcatcgggttaggagagtg |
| pGreen-SGDpro-241mu-F | Dual LUC | gatacattttttgaataactatacctaacaaattccttagac |
| pGreen-SGDpro-241mu-R | Dual LUC | tattcaaaaaatgtatcttatggatttaccgctgt |

Table S2. ^1^H NMR chemical shifts (δ) and coupling constants (Hz) of identified metabolites based on ^1^H-NMR, *J*-resolve, COSY, HSQC and references

| **Compounds** | **Chemical shifts (δ)** |
| --- | --- |
| Isoleucine | 0.96 (t, J = 7.4), 1.03 (d, J = 6.8) |
| Leucine | 0.97 (d, J = 6.3), 0.99 (d, J = 6.3) |
| Valine | 1.01 (d, J = 7.0), 1.06 (d, J = 7.0), 2.28 (m) |
| Threonine | 1.34 (d, J = 6.6) |
| Alanine | 1.48 (d, J = 7.2) |
| Arginine | 1.70 (m), 1.90 (m) |
| Glutamic acid | 2.04 (m), 2.12 (m), 2.39 (m) |
| Glutamine | 2.15 (m), 2.48 (m) |
| Aspartic acid | 2.64 (dd), 3.83 (dd) |
| Asparagine | 2.82 (dd, J = 16.9, 8.2), 2.96 (dd, J = 16.9, 3.9) |
| Serine | 3.78 (dd, J = 6.2, 3.7), 3.92 (dd, J = 12, 6.2), 3.98 (dd, J = 12, 3.7) |
| 2,3-butanediol | 1.15 (d, J = 6.4), 3.50 (m) |
| EtOH | 1.19 (t, J = 7.1) |
| Quinic acid | 1.88 (dd), 1.93 (m) |
| Lactic acid | 1.37 (d, J = 7.2) |
| Acetic acid | 1.94 (s) |
| Malic acid | 2.45 (dd, J = 15.6, 7.2), 2.72 (dd, J = 15.6, 3.9) |
| Citric acid | 2.44 (d, J = 15.6), 2.71 (d, J = 15.6) |
| Ketoglutaric acid | 3.00 (t, J = 7.5) |
| Succinic acid | 2.51 (s) |
| Oxalacetic acid | 3.65 (s) |
| Fumaric acid | 6.55 (s) |
| Sucrose | 5.41 (d, J = 3.8) |
| α-glucose | 5.18 (d, J = 3.7) |
| β-glucose | 4.58 (d, J = 7.9) |
| Choline | 3.21 (s) |
| Chlorogenic acid | 7.61 (d, *J* = 15.9), 7.14 (d, *J* = 2.1), 7.05 (dd, *J* = 8.4, 6.36 (d, *J* = 15.9) |
| 4-O-Caffeoyl quinic acid | 7.67 (d, *J* = 15.9), 7.17 (d, *J* = 2.1), 7.08 (dd, *J* = 8.3, 2.0), 2.1), 6.44 (d, *J* = 15.9), 2.09 (m) |
| 2,3-DHBA | 6.83 (t, J = 8.0), 7.26 (dd, J = 8.1, 1.5), 7.52 (dd, J = 7.9, 1.5) |
| Quercetin-3-O-glucoside | 7.87 (d, J = 2.1), 7.65 (dd, J = 8.5, 2.1), 6.99 (d, J = 9.3), 6.31 (d, J = 2.1), 6.50 (d, J = 2.1), 5.33 (d, J = 6.8) |
| Kaempferol 1 | 6.30 (d, J = 2.1), 6.51 (d, J = 2.1), 7.00 (d, J = 9.3), 8.04 (dd, J = 9.0, 2.2) |
| Kaempferol 2 | 6.30 (d, J = 2.1), 6.51 (d, J = 2.1), 6.98 (d, J = 9.3), 8.08 (dd, J = 8.9, 1.4) |
| Loganic acid | 7.06 (d, *J* = 1.1), 5.30 (d, *J* = 3.2), 4.72 (d, *J* = 8.0), 1.07 (d, *J* = 6.9). |
| Secologanin | 7.44 (dd, J = 11.7, 0.9), 7.56 (d, J = 1.9), 9.65 (d, J = 1.4) |
| Catharanthine | 1.10 (t, J = 7.3), 7.36 (d, J = 8), 7.55 (d, J = 8) |
| Strictosidine | 7.80 (s) |
| Serpentine | 8.31 (d), 8.35 (d), 8.46 (d) |
| Vindoline | 0.51 (t, J = 7.4), 2.00 (s), 2.66 (s), 5.93 (m), 6.22 (d, J = 2.3), 7.11 (d, J = 8) |
| Vindolinine | 1.08 (d, J = 5.6), 5.92 (m), 6.51 (dd, J = 9.9, 3.1), 6.82 (d, J = 7.9), 7.24 (d, J = 7.3) |

Table S3 MIAs and precursors identified in *Catharanthus roseus* by UPLC-Q/TOF MS.

| Compounds | m/z | Identification | Source |
| --- | --- | --- | --- |
| Secologanin | 389.15 | Standard | Sigma-Aldrich |
| Tryptamine | 144.08 | Standard |  |
| Ajmalicine | 353.19 | Standard |  |
| Catharanthine | 337.19 | Standard |  |
| Vinblastine | 406.21 | Standard |  |
| Vindoline | 457.24 | Standard | Shanghai R&D Center for Standardization of Chinese Medicines |
| Anhydrovinblastine | 397.20 | Standard |  |

Table S4 Statistical analysis for selected signals from the NMR spectrum of ethylene treated, MeJA treated and control samples.

|  | ETH-3h | | ETH-24h | | MJ-3h | | | MJ-24h | | |
| --- | --- | --- | --- | --- | --- | --- | --- | --- | --- | --- |
| Metabolites | Fold change | *p* value | Fold change | *p* value | Fold change | *p* value | Fold change | | | *p* value |
| Secologanin | 1.19 | 0.006 | 1.06 | 0.116 | 1.02 | 0.643 | | 1.05 | 0.252 | |
| Strictosidine | 3.08 | 0.006 | 2.33 | 0.016 | 2.25 | 0.031 | | 1.25 | 0.158 | |
| Serpentine | 1.38 | 0.055 | 2.00 | 0.047 | 1.38 | 0.055 | | 1.50 | 0.057 | |
| Vindoline | 1.18 | 0.209 | 1.02 | 0.978 | 1.29 | 0.048 | | 1.04 | 0.898 | |
| Vindolinine | 1.36 | 0.024 | 1.32 | 0.024 | 1.39 | 0.038 | | 1.18 | 0.067 | |
| Catharanthine | 1.07 | 0.101 | 1.15 | 0.016 | 1.11 | 0.055 | | 1.02 | 0.519 | |
| Loganic acid | 1.33 | 0.423 | 1.67 | 0.184 | 0.67 | 0.423 | | 1.00 | 0.900 | |
| Quercetin | 0.93 | 0.047 | 0.91 | 0.189 | 0.84 | 0.016 | | 1.02 | 0.643 | |
| Kaempferol | 0.95 | 0.152 | 0.88 | 0.050 | 0.90 | 0.070 | | 0.89 | 0.013 | |
| 2,3-DHBA | 1.39 | 0.000 | 1.23 | 0.007 | 1.41 | 0.000 | | 1.49 | 0.004 | |
| Fumaric acid | 0.87 | 0.083 | 0.85 | 0.065 | 0.76 | 0.045 | | 0.55 | 0.001 | |
| 4-O-Caffeoyl quinic acid | 0.95 | 0.492 | 1.02 | 0.725 | 0.90 | 0.148 | | 1.02 | 0.667 | |
| Chlorogenic acid | 1.44 | 0.042 | 1.06 | 0.731 | 1.04 | 0.808 | | 1.26 | 0.295 | |
| Sucrose | 0.98 | 0.927 | 1.03 | 0.880 | 0.80 | 0.336 | | 0.87 | 0.487 | |
| α-glucose | 1.42 | 0.109 | 0.29 | 0.015 | 0.72 | 0.199 | | 0.58 | 0.101 | |
| β-glucose | 1.58 | 0.049 | 0.58 | 0.076 | 0.77 | 0.268 | | 0.80 | 0.457 | |
| Oxalacetic acid | 0.77 | 0.236 | 0.93 | 0.645 | 0.76 | 0.218 | | 0.87 | 0.441 | |
| Choline | 1.20 | 0.005 | 1.28 | 0.017 | 1.17 | 0.006 | | 1.23 | 0.046 | |
| Ketoglutaric acid | 1.67 | 0.082 | 0.57 | 0.171 | 1.42 | 0.241 | | 1.10 | 0.760 | |
| Asparagine | 0.86 | 0.155 | 1.34 | 0.087 | 1.04 | 0.812 | | 1.33 | 0.022 | |
| Malic acid | 1.06 | 0.184 | 1.26 | 0.024 | 1.21 | 0.010 | | 1.07 | 0.363 | |
| Citric acid | 0.95 | 0.012 | 0.95 | 0.295 | 1.05 | 0.070 | | 1.12 | 0.001 | |
| Succinic acid | 1.33 | 0.008 | 0.15 | 0.000 | 0.80 | 0.446 | | 0.37 | 0.006 | |
| Glutamic acid | 0.82 | 0.010 | 1.06 | 0.205 | 0.88 | 0.023 | | 1.15 | 0.155 | |
| Glutamine | 1.19 | 0.000 | 1.16 | 0.008 | 1.04 | 0.556 | | 1.07 | 0.003 | |
| Quinic acid | 1.15 | 0.115 | 0.93 | 0.333 | 1.08 | 0.299 | | 1.11 | 0.145 | |
| Arginine | 1.13 | 0.000 | 1.13 | 0.021 | 1.13 | 0.001 | | 1.20 | 0.002 | |
| Alanine | 1.69 | 0.001 | 1.47 | 0.001 | 1.50 | 0.002 | | 1.48 | 0.004 | |
| Lactic acid | 0.95 | 0.612 | 1.11 | 0.327 | 0.94 | 0.710 | | 1.01 | 0.914 | |
| Threonine | 1.08 | 0.329 | 1.03 | 0.754 | 1.33 | 0.020 | | 1.03 | 0.723 | |
| Ethanol | 0.67 | 0.008 | 0.57 | 0.002 | 0.64 | 0.007 | | 0.85 | 0.061 | |
| 2,3-butanediol | 0.92 | 0.003 | 0.85 | 0.014 | 0.84 | 0.000 | | 0.99 | 0.800 | |
| Valine | 1.06 | 0.733 | 1.14 | 0.431 | 0.94 | 0.736 | | 1.12 | 0.604 | |
| Leucine | 1.62 | 0.035 | 1.73 | 0.013 | 1.67 | 0.027 | | 1.30 | 0.344 | |

Statistically significant differences using Student’s t-test: *p* < 0.05.

Fold change: the relative intensity ratio of the treated samples to the control samples (CK-0h).
